# *Mycobacterium tuberculosis* PE/PPE proteins enhance the production of reactive oxygen species and formation of neutrophil extracellular traps

**DOI:** 10.3389/fimmu.2023.1206529

**Published:** 2023-08-22

**Authors:** María García-Bengoa, Marita Meurer, Matthias Stehr, Ayssar A. Elamin, Mahavir Singh, Wulf Oehlmann, Matthias Mörgelin, Maren von Köckritz-Blickwede

**Affiliations:** ^1^ Institute of Biochemistry, University of Veterinary Medicine Hannover, Hannover, Germany; ^2^ Research Center for Emerging Infections and Zoonosis (RIZ), University of Veterinary Medicine Hannover, Hannover, Germany; ^3^ LIONEX Diagnostics and Therapeutics GmbH, Braunschweig, Germany; ^4^ UGA Biopharma GmbH, Hennigsdorf, Germany; ^5^ Colzyx AB, Lund, Sweden

**Keywords:** *Mycobacterium tuberculosis*, *Mtb*, PE18, PE31, PPE26, neutrophil extracellular traps (NETs), reactive oxygen species, therapeutics

## Abstract

**Introduction:**

Neutrophil granulocytes predominate in the lungs of patients infected with *Mycobacterium tuberculosis* (*Mtb*) in earlier stages of the disease. During infection, neutrophils release neutrophil extracellular traps (NETs), an antimicrobial mechanism by which a DNA-backbone spiked with antimicrobial components traps the mycobacteria. However, the specific mycobacterial factors driving NET formation remain unclear. Proteins from the proline-glutamic acid (PE)/proline-proline-glutamic acid (PPE) family are critical to Mtb pathophysiology and virulence.

**Methods:**

Here, we investigated NET induction by PE18, PPE26, and PE31 in primary human blood-derived neutrophils. Neutrophils were stimulated with the respective proteins for 3h, and NET formation was subsequently assessed using confocal fluorescence microscopy. Intracellular ROS levels and cell necrosis were estimated by flow cytometry. Additionally, the influence of phorbol-12-myristate-13-acetate (PMA), a known NADPH oxidase enhancer, on NET formation was examined. Neutrophil integrity following incubation with the PE/PPE proteins was evaluated using transmission electron microscopy.

**Results:**

For the first time, we report that stimulation of primary human blood-derived neutrophils with *Mtb* proteins PE18, PPE26, and PE31 resulted in the formation of NETs, which correlated with an increase in intracellular ROS levels. Notably, the presence of PMA further amplified this effect. Following incubation with the PE/PPE proteins, neutrophils were found to remain viable and structurally intact, as verified through transmission electron microscopy, indicating the occurrence of vital NET formation.

**Discussion:**

These findings offer valuable insights that contribute to a better understanding of host-pathogen interactions during *Mtb* infection. Moreover, they underscore the significance of these particular *Mtb* antigens in triggering NET formation, representing a distinctive and previously unrecognized function of PE/PPE antigens.

## 1 Introduction

*Mycobacterium tuberculosis* (*Mtb*) is the causative agent of tuberculosis (TB), an infectious disease that results in a lasting, important public health threat causing approximately 1.6 million deaths worldwide in 2021, 187.000 of whom were additionally infected with HIV (1). The relationship of *Mtb* with the host immune system is not yet fully understood. However, a better elucidation of the interaction between the host and the pathogen is necessary for developing new strategies against *Mtb*.

Neutrophils are known to contribute to the control of TB infection by several bactericidal mechanisms including phagocytosis, degranulation, oxidative killing by reactive oxygen species (ROS), and the release of neutrophil extracellular traps (NETs) (2, 3). The formation of NETs is an extracellular mechanism of neutrophils to entrap and partially kill microbes (4). NETs are fibrous networks made of chromosomal DNA, histones, antimicrobial peptides, and granule proteins that can capture the mycobacteria. Thus, the activation of neutrophils and subsequent NET formation is thought to be an immune response aimed at containing and eliminating the bacteria (4). Neutrophils release NETs *in vitro* as a response to *M. tuberculosis* and also *in vivo* in the circulation of patients with active TB (ATB) (5–9). According to a study by Schechter et al., increased NETs showed by levels of neutrophil elastase (NE) and myeloperoxidase (MPO), correlated with disease severity in plasma from TB patients (10). Similarly, NET-associated MMP-8 is increased in sputum from TB individuals (11). Higher levels of neutrophilia-related serum cit-H3, another NET marker (12, 13), were also found in lung tissue damage patients correlating with increased cavity formation (8). Overall, NETs seem to play a central role during TB, and therefore a better understanding of the implications of NET-formation during disease would be useful to develop new therapeutics. As described, two different pathways can lead to the formation of NETs containing nuclear DNA (4, 14–16). Those are “suicidal” NETosis (also referred to as “classical” NETosis) (4, 14) and “vital” NET release (15, 16). During the classical NETosis pathway, specific transduction signal cascades are activated leading to the active form of the NADPH oxidase (NOX2) (nicotinamide adenine dinucleotide phosphate oxidase) and subsequent release of ROS, as induced for example by phorbol-12-myristat-13-acetat (PMA), a classical activator of NETosis. As for the vital mechanism, some authors state that NETs are released via ROS-independent mechanisms (15, 17). However, a study by Yousefi et al. showed that neutrophils can release mitochondrial DNA (mtDNA) through vesicles dependent on superoxide production along with granulocyte-macrophage colony-stimulating factor (GM-CSF)-mediated priming of neutrophils (18). Importantly, whereas NETosis is a cell death mechanism, early NET release from intact cells is also possible, allowing cells to perform other cellular functions such as phagocytosis (15). During the “vital” NET release, the morphology of the nucleus experiences important changes with separation between the inner and the outer nuclear membranes, and nuclear vesicles are released into the cytoplasm. However, the nuclear membranes and nuclear pore complexes remain intact as revealed by transmission electron microscopy (15). Additionally, intact cells can be found surrounded by extracellular DNA-NETs. If NETs play a beneficial or detrimental role in the host during TB infection is still unclear, thus requiring further investigation. A better understanding of the mechanisms leading to the formation of NETs in response to *Mtb* is an important step to additionally characterize its relevance as a therapeutic target.

Interestingly, Francis et al. found the early secreted antigenic target protein-6 (ESAT-6), an ESAT-6 secretion system 1 (ESX1) antigen that is present in pathogenic *Mtb*, to cause necrosis in neutrophils and possibly NETs (19), whereas, in a different study, *Mtb* was shown to induce the formation of macrophage extracellular traps depending on the ESX1 gene cluster (20). Altogether, ESX1 seems to play an important role in the pathogenesis of *Mtb* through the release of ETs. However, the vaccination strain BCG lacking ESX1 was also shown to induce the formation of NETs (21). ESX5, the most recent ESX cluster that is only present in slow-growing species of mycobacteria, is responsible for the secretion of the majority of proline-glutamic acid (PE)/proline-proline-glutamic acid (PPE) proteins of *Mtb* (22–24). PE/PPE proteins directly encoded within the *esx5* locus: PPE25 (Rv1787), PE18 (Rv1788), PPE26 (Rv1789), PPE27 (Rv1790), and PE19 (Rv1791), represent potential virulence factors as shown by the attenuation of a *Mtb* deletion mutant lacking the five *esx5-*associated *pe* and *ppe* genes (25). Furthermore, PE31 (Rv3477) is associated with the *esx5* gene cluster due to its high homology and genomic orientation (22), and is considered a virulence factor of *Mtb* (26). Interestingly, PE/PPE proteins can cross-react with components from a different cluster (27, 28), and many of these *pe* genes are adjacent to *ppe* genes being co-expressed (29, 30). Thus, the resultant complexes or heterodimers will be exposed on the cell surface or secreted, allowing them to directly interact with the host (31–35).

In order to characterize *Mtb* antigens it is necessary to understand the interaction and connection between the host and the pathogen. Based on the literature and online databases, Tuberculist, Mycobrowser, SWISS-model, and String v11 (36–39), we selected three *Mtb* antigens that have been predicted or experimentally shown to induce immune responses and belong to the PE/PPE family. Thus, the ESX5-associated proteins PE18 and PPE26 were chosen as well as the PE31 candidate, the exact roles of which in the host-pathogen interactions and underlying mechanisms remain unknown. In the present study, we aim to elucidate if non-ESX1 encoded proteins PE18, PE31, and PPE26 induce the formation of ROS, and if this is associated with an enhanced formation of neutrophil extracellular traps.

## 2 Materials and methods

### 2.1 Cloning of *Mycobacterium tuberculosis* genes encoding PE/PPE candidates

Synthetic genes PE18, PPE26, and PE31 were obtained from *Eurofins* (Ebersberg, Germany*).* Codon optimization was applied before including the corresponding restriction sites and 6xHis-tag was added to the PPE26 gene at the N-terminus. PE18 and PE31 genes were later modified by adding His-tags at the C-terminus by PCR using specific primers (**Table S1**).

The PPE26 gene was subjected to double digestion with *NdeI* and *EcoRI* restriction enzymes and ligated into the pWO1022 cloning vector (LIONEX GmbH, Germany). Additionally, PE18 and PE31 genes were cloned into plasmidic vectors pETDuet™-1(MCS-1) and pET28a (both Novagen) respectively, by restriction enzymes *NcoI* and *HindIII*. Then, the linearized vectors and inserts were extracted from the agarose gel by using a gel purification kit (Qiagen). The T4 DNA ligase (Thermo Scientific) was engaged for the ligation of the PE and PPE genes inserted into the correspondent vectors resulting in the following recombinant expression plasmids: pETDuet-1-PE18, pET28a-PE31, and pWO1022-PPE26. Recombinant constructs were used to transform *Escherichia coli BL21-DE3* (Invitrogen) strain. Transforming single colonies were selected on Luria Broth (LB) plates containing the correspondent antibiotic, 50μg/mL Kanamycin (Roth) or 400μg/mL Ampicillin (Sigma Aldrich), and screened by colony PCR amplification with specific primers (**Table S2**) for T7 promoter and the products were analyzed on a 1% agarose gel. The recombinant plasmids from the positive PCR colonies were purified using a Mini Spin Prep kit (Qiagen) and double-digested for confirmation.

### 2.2 Expression and purification of recombinant proteins

Cells were grown in LB broth, containing 50μg/mL Kanamycin or 400μg/mL Ampicillin supplemented with 1% Glucose, and incubated at 37°C on a 130 RPM shaker until turbidity of 0.8 at A600nm was reached. For the expression of recombinant proteins, cultures were induced with 1mM Isopropyl β-D-1-thiogalactopyranoside (IPTG) for 3h and overnight at 37°C shaking. The overnight cultures were harvested by centrifugation at a speed of 5826xg at 20°C for 15 min and the resultant supernatants were discarded. Purification of recombinant proteins was carried out by sonicating bacterial cells for 30 seconds 6 times at 80% amplitude with one minute pause between cycles, and solubilizing recombinant proteins using 20mM Tris, 100mM NaCl 1% Triton X-100, pH 8.0 buffer. To reconstitute proteins from inclusion bodies, pellets were washed in 20mM Tris, 100mM NaCl, and 1% Triton X-100 pH 8.0 twice, as well as an additional washing step in 20mM Tris, 100mM NaCl, pH 8.0. Inclusion bodies were later denatured when resuspended in 20mM Tris, 100mM NaCl, 8M Urea pH 8.0. His-tag recombinant proteins were purified by Ni-NTA affinity chromatography using an ÄKTAprime plus device (GE Healthcare), and eluted with 500mM Imidazole. Fractions with the protein of interest were pooled and examined on a 15% SDS-PAGE performed with a Hoefer Scientific SE250 vertical protein electrophoresis unit (Hoefer Scientific 84 October Hill Road Holliston, MA 01746-1388 USA), and followed by Coomassie Brilliant Blue staining. Protein gels were analyzed with a Bio-Rad Gel Doc EZ automated imaging system and Image Lab software version 5.0 (Bio-Rad Bio-Rad Laboratories GmbH, Kapellenstraße 12, D-85622 Feldkirchen Germany). Eluted proteins were loaded onto a Sephadex G-25 column (Sigma-Aldrich) pre-equilibrated in endotoxin-free 10mM NH_4_HCO_3_ pH 8.0 and refolded. Endotoxins were removed during the purification process by the addition of 1% Triton X-100. Purified proteins had minimal final endotoxin content (<10 EU/mL), as measured by the *Limulus amebocyte lysate* (LAL) test (PYROTELL^®^-T). Protein concentration was determined by using the Lowry assay (Biorad) analyzed in NanoPhotometer (7122 V2.1.0, 2035) for protein quantification. All proteins were stored in three different conditions: 4°C, -20°C, and lyophilized in 10mM NH_4_HCO_3_ pH 8.0 for later stability tests.

### 2.3 PE18, PE31, and PPE26 detection in western blot analysis

The same amount of PE18, PE31, and PPE26 (5µg) was separated and analyzed by SDS-PAGE using a vertical double gel system PEQL45-1010 (PEQLAB; VWR international, Hilpertstraße 20a, 64295 Germany), and an in-house positive control protein was also included. Gel electrophoresis was performed with 10-20% Tris-tricin-gels (TR120 12) (anamed Elektrophorese GmbH, Ringstraße 4, 64401 Groß-Bieberau/Rodau, Germany). After electrophoresis, proteins were transferred onto a PVDF membrane (Millipore) for 11 min at 25V 2.5mA with a Trans-Blot Turbo Transfer System (Biorad). For transfer, 1x Trans-Blot Turbo transfer buffer was used (diluted according to manufacturer’s instructions from 5x Transfer Buffer (Biorad). The membrane was blocked in 0.05% TBS-Tween 1% BSA for 1h at room temperature. For the detection of the recombinant proteins, the blot was incubated with the polyclonal mouse anti-His (Qiagen) antibody for 2h at room temperature with agitation. Additionally, a polyclonal rabbit anti-*E.coli BL21 (DE3)* (LIONEX GmbH, Germany) antibody was used as the control for 1h at room temperature with agitation. The respective secondary antibodies (goat anti-mouse IgG-HRP and goat anti-rabbit IgG-HRP) (Thermo Scientific) were added for 1h and 30 min at RT with agitation. Proteins were detected using TMB-membrane for 15 minutes and respective signals were found at the expected sizes. Protein identification by Mass Spectometry was carried out through an external service (*Proteome Factory AG*, Berlin), by preparation of the samples in a 15% SDS-PAGE, and further processing after cutting out the protein bands from the destained SDS polyacrylamide gels. Furthermore, protein identity was confirmed by N-terminal Edman sequencing after the three proteins were transferred into a PVDF membrane. Five N-terminal amino acid sequencing steps were performed using the Procise method (*Proteome Factory AG*, Berlin).

### 2.4 Polyclonal antiserum generation in rabbit against purified PE/PPE recombinant proteins

Serum from immunized rabbits containing polyclonal antibodies against PE and PPE proteins of interest was generated and provided by *Charles River* (France). Briefly, on days 0, 28, 42, and 56, rabbits (NZW) were immunized subcutaneously with purified PE18, PE31, or PPE26 protein together with aluminum used as an adjuvant. Before immunization, blood was collected from rabbits, and correspondent serum was used as the negative control. On day 70, rabbits were bled and serum was collected and stored at −80°C until used.

### 2.5 Antigen-specific antibody titers

Antigen-specific serum IgG antibody responses in serum samples drawn from immunized rabbits were evaluated by in-house ELISA. Next, 96-well microtiter plates were coated overnight at 4°C with two different concentrations of each recombinant protein (2 and 4μg/mL) in duplicate in 10mM ammonium bicarbonate pH8 coating buffer. Afterward, plates were washed three times with PBS pH 7.5 0.5% Tween-20 (PBS-T) and blocked with PBS pH 7.4 1% BSA blocking solution for 2h at 37°C. Plates were washed again and stored at 4°C under dry conditions until the collection of polyclonal serum from immunized rabbits. Later, plates were incubated with rabbit polyclonal serum in 3-fold serial dilutions (1:50 starting dilution) for 1h RT and underwent repeated washes before the addition of secondary antibody (1:5000) (horseradish peroxidase (HRP)-conjugated goat anti-rabbit IgG) (Thermo Scientific). Plates were washed again and incubated with TMB (Thermo Scientific) for 20 minutes. The resultant reaction was halted with a stop solution for TMB substrate (Thermo Scientific).

### 2.6 Protein-protein affinity studies

Structures of PE18 (and PE31) in complex with PPE26 were predicted by homology modeling using the solved crystal structure of PE8-PPE15 as the template by the SWISS-MODEL template library. To further elucidate the interaction between the PE and PPE candidates, the binding kinetics of PE18-PPE26 and PE31-PPE26 were analyzed by using biolayer interferometry and the *ForteBio* Octet QK Biosensor platform. For binding evaluation (3-aminopropyl) triethoxysilane (APS) biosensors (LOT No.:1511162) were used. All steps were performed at a shake rate of 1000rpm and a temperature of 20°C, except for the first base line and the loading steps, which were performed at a shake rate of 800rpm. Samples were prepared on a dark microtiter plate (96-well) and biosensors were equilibrated with PBS for 180 seconds as establishing the baseline. Afterward, biosensors were loaded with antigen PE18 (31.33µM) or PE31 (46.12µM) as the ligand for 1800 seconds and later dipped onto 0.05% BSA for 300 seconds. A second baseline was established by loading biosensors with 10mM NH_4_HCO_3_ buffer for 260 seconds. For the association phase, biosensors were dipped into the PPE26 analyte 1:2 dilution series (stock solution 1mg/mL) for 3600 seconds, followed by the dissociation step, in which biosensors were dipped for 1500 seconds into 10mM NH_4_HCO_3_ buffer. At the end, biosensors were regenerated and washed by dipping into 1% Triton X-100 (Sigma-Aldrich) and 1x PBS subsequently. By using the *Octet Data Analysis software*, the raw data acquired for the interaction between the PEs with protein PPE26 was processed and fit to a curve in order to extract values of k_on_, k_diss,_ and K_D_. The ratio of k_off_ to k_on_ determines the K_D_ reported here. For data processing, reference correction was performed to compensate for the signal drift of the immobilized biosensor with the assay buffer (unloaded biosensor). Subsequently, Y-axis alignment to baseline, inter-step correction, and Savitzky-Golay filtering were applied to increase the precision of the data without distorting the signal.

### 2.7 Human neutrophil isolation

Blood samples were drawn from healthy donors by a physician, in agreement with the local ethical board. The study was approved by the ethical committee of the Hannover Medical School Nr. 3295-2016. Human neutrophils were isolated and purified from fresh heparinized venous blood by density gradient centrifugation at 480xg using Polymorphprep™ (Axis-Shield, PoC) as previously described (40). The neutrophil-rich cell band was collected and washed with PBS. Neutrophil viability was >95% after 0.4% trypan blue (ROTH) exclusion for all preparations. Finally, cells were resuspended in 1mL RPMI 1640 medium (Gibco) and the cell number required for the experiments was adjusted with RPMI accordingly.

### 2.8 Quantification of intracellular ROS and cell death

The levels of intracellular ROS were measured using the cell-permeable oxidation-sensitive fluorescent dye H2DCF-DA (2, 7-dichlorodihydrofluorescein diacetate, Invitrogen, Carlsbad, CA, USA) and propidium iodide (PI) (Sigma-Aldrich, St. Louis, USA) was used for cell death determination. Briefly, 1×10^5^ neutrophils were seeded in 200μL RPMI 1640 medium per well in a 96-well-plate and stimulated with all three PE/PPE candidates, as single proteins and in all possible combinations, at respective concentrations of 1, 5, and 10µg/mL for 3h. Wells containing only cells (and no stimulatory recombinant protein) served as unstimulated controls, whereas stimulation with 25nM PMA (Sigma–Aldrich), the potent inducer of typical NETs, served as positive control. Furthermore, ESAT-6 and CFP-10 (LIONEX GmbH, Germany) proteins of *Mtb* were used as control proteins. Afterward, cells were loaded with 10μM of the H2DCF-DA dye and 0.25μg/mL PI, and incubated for 3h at 37°C. ROS and PI levels were analyzed immediately by Fluorescence-activated cell scanning (FACS) by using the Attune NxT Cytometer (Thermofisher) with the following setup: the threshold was adjusted to heat-killed cells for PI and control live cells for H2DCF-DA positive cells. Acquisition volume was set to 50µL and acquisition speed was set to 100µL/min. FSC and SSC settings were optimally adjusted to the size and granularity of neutrophil granulocytes. ROS was detected with the BL1 530/30 filter of the blue laser (488nm) and PI with the YL1 585/16 filter of the yellow laser (561nm). Fluorescence intensity was analyzed using FlowJo software version (v)10. For the determination of living and dead cells, gates were set with regard to the dead control. All FACS analyses in one experimental set-up were analyzed with the same gate. **Supplementary Figure 1** shows the gating strategy set for a mixed population of live and dead cells, which was later applied to all FACS analyses. Each experiment was performed in duplicates and in the presence or absence of PMA. The complete experiment was repeated five times with neutrophils of different blood donors for statistical purposes.

### 2.9 Stimulation of neutrophils with PE/PPEs for confocal microscopy and calprotectin detection

A total of 2×10^5^ cells in a 200μL RPMI 1640 final volume (phenol red free, PAA) were seeded per well in a 48-well-plate on Poly-L-lysine (Sigma-Aldrich) coated 8mm glass coverslips (Epredia, Netherlands). Neutrophils were respectively stimulated with 25nM PMA (Sigma–Aldrich), 10µg/mL of the proteins PE18, PPE26, or PE31, or left unstimulated, and plates were immediately centrifuged for 5 min at 250xg to sediment neutrophils and NETs. Cells were incubated for 3h at 37°C with 5% CO_2_. After incubation, the cells were centrifuged for five minutes at 250xg RT, fixed with 4% PFA (Science services) for 15 min at room temperature, and kept at 4°C until subsequent immunostaining after washing. Supernatants from each well were collected before fixation and used for estimation of levels of calprotectin (Human calprotectin ELISA Kit, MyBioSource) according to manufacturer instructions in a microtiter plate reader (Multiskan FC, Thermo Scientific).

### 2.10 Immunostaining of NET-specific markers for visualization of NETs

Fixed cells were washed three times with 1x PBS, permeabilized with 0.5% Triton X-100 PBS for 5 min at room temperature, and blocked by using 5% goat serum (Sigma) in 0.5% Tween PBS for 20 minutes at room temperature. Incubation with a mouse monoclonal IgG2a anti-DNA/histone (Millipore, Billerica, Massachusetts, USA) and a rabbit anti-human myeloperoxidase (Dako) in 5% goat serum 0.5% Tween PBS was carried out for 1h at room temperature. Isotype controls, murine myeloma IgG2a and IgG from rabbit serum (both from Sigma) were included to avoid unspecific signals. Samples were later washed with PBS three times and subsequently incubated with an Alexa-Fluor-488-labelled goat-anti-mouse IgG2a antibody, and an Alexa-Fluor-633-labelled goat-anti-rabbit IgG, for 45 min at room temperature in the dark. After washing, slides were mounted in ProlongGold^®^ antifade with DAPI (Invitrogen), and cover slips were surrounded with clear nail polish and stored at 4°C in the dark until confocal fluorescence microscopy analysis.

### 2.11 Visualization and quantification of NETs by confocal laser scanning microscope

NETs were observed by using a Leica TCS SP5 confocal microscope with an HCX PL APO 40x 0.75–1.25 oil immersion objective. Settings were adjusted in accordance to control preparations using the isotype control antibody. Six randomly chosen pictures were taken from each sample and only in the case of an artifact present (e.g., air bubble), a different adjacent area was selected. When taking the pictures, the focus was set on the nuclei (blue channel). Percentages of NET-releasing cells were calculated using ImageJ free-software (version 1.52q; National Institute of Health, Bethesda, MD, USA) and counting cells with the Cell counter plugin from 3 pictures taken from each sample in duplicates. Then, the NET release was determined as a percentage of NET-forming cells (NET-activated cells) to the ones without NETs. An average from the counted NET-activated cells from the six pictures from each sample was calculated. A neutrophil was counted as positive when an evident off-shoot of DNA was visible being released by that exact cell or in contact with cells in the surrounding area, or if at least two of the following criteria were found: enlarged nucleus, decondensed nucleus, or blurry rim.

### 2.12 Electron microscopy of neutrophils stimulated with PE31

A total of 1×10^6^ cells in 1mL RPMI 1640 final volume (phenol red-free, PAA) were seeded in a 1.5mL tube. The cells were stimulated with either 25nM PMA (Sigma–Aldrich), or 10µg/mL of PE31 for 3h at 37°C with 5% CO_2_. After incubation, the cells were centrifuged for five minutes at 400xg RT, and resultant pellets were fixed with 250µL of 2.5% glutaraldehyde (Sigma) in 0.1M sodium cacodylate (pH 7.2) buffer and kept at 4°C until subsequent transmission electron microscopy and immunostaining. Supernatants were collected before fixation and frozen at -20°C.

For transmission electron microscopy, the fixed and washed samples were subsequently dehydrated in ethanol and further processed for standard Epon embedding, as described previously (41, 42). Specimens were cut in 70nm ultrathin sections with an LKB ultratome and mounted on Formvar-coated copper grids. The ultrathin sections were stained with uranyl acetate and lead citrate (both Laurylab). The immunolabeling of thin sections after antigen unmasking with sodium metaperiodate (Merck) (43) was performed as described previously (44), with the modification that Aurion-BSA (Aurion) was used as a blocking agent. The following antibodies were used: gold-labeled Anti-Histone H3 (citrulline R2 + R8 + R17) antibody (1:80 diluted; H3cit, 5 nm gold; Abcam), anti-neutrophil elastase (1:80 diluted; NE, 10 nm gold; Abcam) and gold-labeled anti-PE31 polyclonal rabbit antibody (1:50; PE31 15 nm gold). The images were recorded using a Philips/FEI CM100 BioTwin transmission electron microscope operated at a 60kV accelerating voltage, with a Gatan Multiscan 791 charge-coupled device camera.

### 2.13 NET quantification in EM images

For the quantification of NETs, the cellular profiles from 30 fields were selected randomly on the thin sections and analyzed per sample for two different analyses that are listed below (2.13.1 and 2.13.2). For each analysis, 30 different fields were assessed as follows:

#### 2.13.1 NET release from the nuclei

Loss of intact cytoplasmic structure with release of nuclear DNA and presence of nuclear vesicles in the surrounding containing double-positive staining for NE and H3cit, was counted as positive. The percentage of positive cells was calculated in relation to all of the observed cells investigated.

#### 2.1.3.2 Nuclear vesicles per neutrophil

The number of nuclear vesicles (H3cit and NE positive) was counted per neutrophil in the randomly selected fields. The analyzed neutrophils show an intact cytoplasmic structure with a nucleus, double nuclear membrane, and intact granules.

### 2.14 Statistical analysis

For statistical analysis and graphing, GraphPad Prism 9.0 (Graph Pad Software) was used. Data derived from a minimum of three independent experiments were analyzed and presented as the mean ± SD. For all graphs shown, except for the quantification of vesicular NETs in the EM images, one-way ANOVA with matched measures followed by Dunnett’s multiple comparison test was used, as indicated. Sphericity was applied since the variability of differences among groups was considered equal. The differences between the groups were analyzed, as described in the figures’ legends (ns p> 0.05, * p < 0.05, ** p < 0.01, *** p < 0.001, **** p < 0.0001). In the case of vital NETs quantification, a paired t-test was used (**** p < 0.0001).

## 3 Results

### 3.1 PE18, PE31, and PPE26 expression

**Figure 1:**
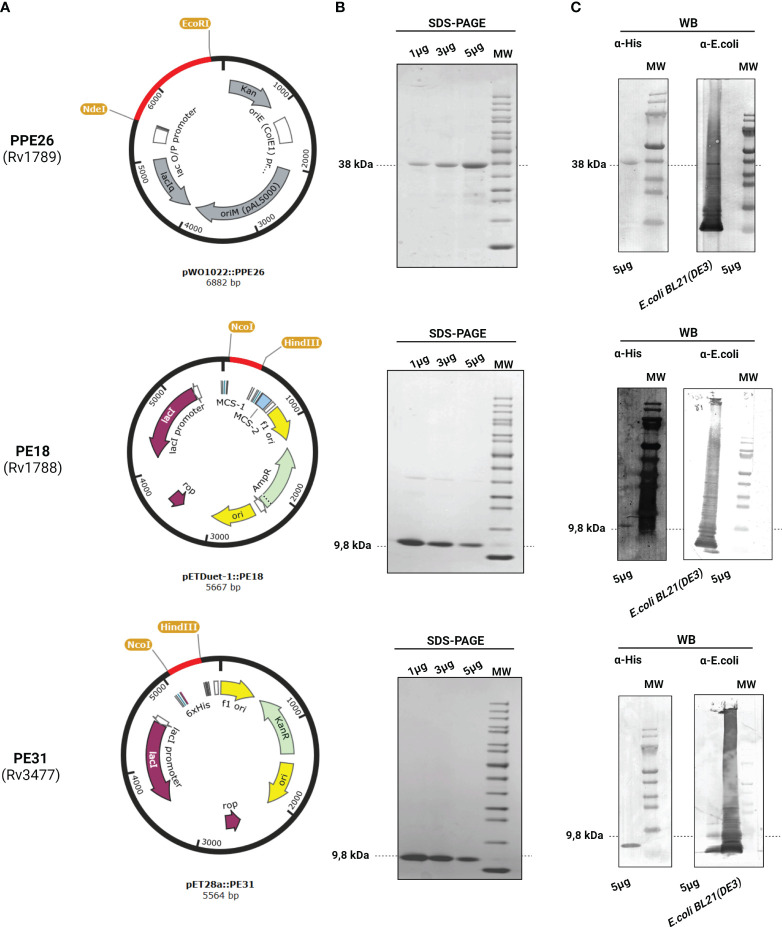
Expression and purification of PPE26 (Rv1789), PE18 (Rv1788), and PE31 (Rv3477) from *Mycobacterium tuberculosis*. **(A)** Schematic representation of the expression vector used for each construct, highlighting the incorporation of the respective genes within the vector backbone. **(B)** Resultant recombinant proteins were identified by SDS-PAGE electrophoresis by loading 1, 3, and 5µg of the proteins. The expected molecular weight of each protein is indicated in bold. **(C)** Corresponding Western Blot (WB) analysis was performed using 5µg of each protein. When incubated with the polyclonal mouse anti-His antibody, the WB membrane exhibits specific bands that correspond to respective proteins PPE26, PE18, and PE31 (5µg) indicated by arrows, confirming successful expression and purification. However, no signal was detected when membranes were incubated with the polyclonal rabbit anti-*E.coli* antibody. Lysate of *E.coli BL21 (DE3)* was used as a positive control for the polyclonal rabbit anti-*E.coli* antibody. Representative images are shown.

To investigate the role of PE18, PE31, and PPE26 proteins in NET formation in human-derived blood neutrophils, the respective genes from *Mtb* H37Rv were synthesized and cloned into the expression vectors pETDuet™-1(MCS-1), pET28a, and pWO1022 respectively (**Figure 1A**), and the vectors were transformed into *Escherichia coli BL21 (DE3)* to express the target proteins with a 6xHis-tag as described under “Materials and Methods”. The His-tag fusion proteins were then purified by nickel affinity chromatography and analyzed by SDS-PAGE (**Figure 1B**) and Western blotting (**Figure 1C**) using a polyclonal mouse anti-His antibody. As shown in **Figure 1C**, the Western blot analysis with anti-His antibody showed the expression of the target proteins at the expected molecular mass (9,8kDa for the PEs and 38kDa for the PPE26 protein). The purified recombinant proteins were further used to study protein-protein interactions and to assess ROS production and NET formation in human neutrophils by using the proteins as individual proteins as well as in combinations to test them as complex forms. PE/PPE proteins are difficult to clone given their high GC content (around 80%) and highly repetitive genetic sequences, and to purify as soluble and stable recombinant proteins due to their high structural disorder and the need for a partner for folding (45–47). Together, we successfully expressed and produced proteins PE18, PE31, and PPE26 as individual soluble recombinant proteins.

### 3.2 PE18-PPE26 and PE31-PPE26 binding affinity suggests complex formation

**Figure 2:**
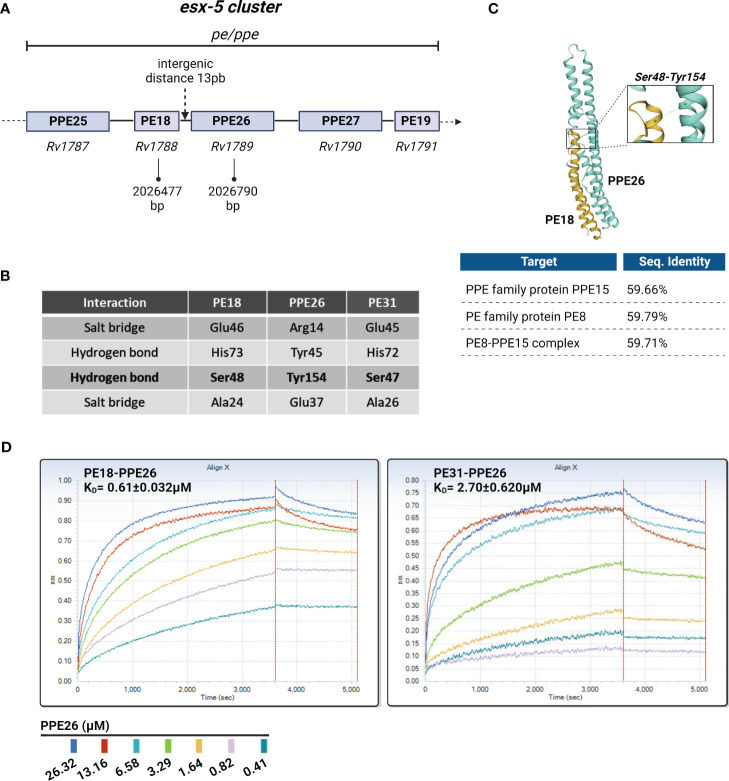
Overview of the putative PE18/31-PPE26 complexes. **(A)** Schematic representation of PE18-PPE26 and other *pe* and *ppe* genes within the ESX5 cluster. PE18 is located upstream (5’) of the PPE26 gene with a minimal intergenic distance of 13 pair bases between the two ORFs, suggesting they form a putative PE/PPE complex. **(B)** Table shows the conserved residues that may participate in protein-protein interactions present in PE18-PPE26 and PE31-PPE26. The Glu46-Arg14 interaction is contained in 60% of PE/PPE complexes to maintain contact between the α2 helix of PE and the α1 helix of PPE, whereas the conserved hydrogen bond Ser48-Tyr154 is critical for minimal binding and stabilizes interactions between the α2 helix of PE and α5 helix of PPE. All interactions mentioned in the table for proteins PE18 and PPE26 have been previously described for other complexes more widely studied like PE8-PPE15 and PE25-PPE41. **(C)** Three-dimensional structure of the PE18-PPE26 complex was created by homology modeling fitting to a PE8-PPE15 complex template computed by the SWISS-MODEL server. Percentages of sequence identity are depicted for the proteins alone and for the overall structure. **(D)** Rapid complex formation evaluation run on the Octet QKe. Data are shown for the loading of PE18 (31.33 µM) or PE31 (46.12 µM) onto an APS surface and after BSA blocking sensors’ exposure to different concentrations of the PPE26 protein. Assay negative control was 0µM PPE26. The association and dissociation steps were processed and fit with a 1:1 binding model. Curve fitting of the processed curves provided k_on_, k_diss_, and K_D_ for the interaction of the complexes.

Because PE18 and PPE26 are localized in the same operon, we hypothesized that PE18 and PPE26 may interact as a complex. Previous bioinformatic approaches predicted PE18 (Rv1788) and PPE26 (Rv1789) to be co-operonic based on the small intergenic distance between the two open reading frames (ORFs) (48) (**Figure 2A**). Only two PE/PPE complex crystal structures have been solved until now being EspG5-PE8-PPE15 (46) and PE25-PPE41 (45, 49). The two complexes share a conserved hydrogen bond between the Tyr154 (PPE) and Ser48 (PE) residues (46) described to be required for minimal binding. Such binding was described to stabilize interactions between the α2 helix of PE and α5 helix of PPE and most probably, is a common property of PE/PPE complexes (46).

First, we did a comparison of PE18-PPE26 with the PE8-PPE15 (strain H37Rv) pair by homology modeling computed by the SWISS-MODEL server, and they exhibit very similar folding characteristics due to their high percentage of coverage (59.66% sequence identity with PPE15 and 59.79% with PE8) (**Figure 2C**). Importantly, numerous conserved residues known to participate in protein-protein interactions among PE and PPE proteins (46) are also conserved in the putative PE18-PPE26 complex, including the conserved hydrogen bond Ser^48^_PE-_Tyr^154^_PPE_. Furthermore, other sites of interaction that have been described as well as variations were additionally identified (**Figure 2B**). Moreover, we investigated if PE31 would have an affinity for PPE26 due to the high percentage of identity between the amino acid sequences of PE18 and PE31 (64% identity). To study if there is an affinity between the two proteins of the respective complexes, we analyzed the binding kinetics of the two pairs using biolayer interferometry technology (**Figure 2D**). The kinetics of association and dissociation for the interaction between PE18-PPE26, and PE31-PPE26 were measured and resultant values for k_on_ (M^-1^s^-1^), k_diss,_ (s^-1^) and K_D_ (M^-1^) were obtained after fitting the data to a 1:1 binding model. The K_D_ values, calculated as the ratio of k_diss_/k_on_, were 0.61 ± 0.032µM for PE18-PPE26 and 2.70 ± 0.62µM for PE31-PPE26, indicating that PE31-PPE26 has a weaker binding affinity compared to the PE18-PPE26 pair. Raw binding data for both experiments produced concentration-dependent binding curves for the analyte (PPE26 antigen) from 26.32 to 0.41µM (**Figure 2D**).

### 3.3 PE/PPE candidates induce the production of intracellular ROS in human neutrophils

We first evaluated if PE18, PE31, and PPE26 are able to enhance the production of ROS in human neutrophils derived from healthy donors. Previous studies have reported the formation of heterodimeric complexes involving PE and PPE proteins, which can influence the resulting immune response (46, 49). To test that, the three candidates were used alone and in all possible combinations. Previously, Rojas et al. demonstrated that a concentration of 10µg/mL of *Mtb* soluble recombinant protein ESAT-6 was effective in inducing visible changes in the nuclear morphology of neutrophils (50). Furthermore, in immunization studies, PE/PPE antigens have been delivered at a concentration of 10µg/mL (51, 52). In this study, we performed dose-response experiments within the range of 1 to 10µg/mL for each specific PE/PPE protein, and neutrophils, to ensure adequate stimulation and observation of the desired effects.

**Figure 3:**
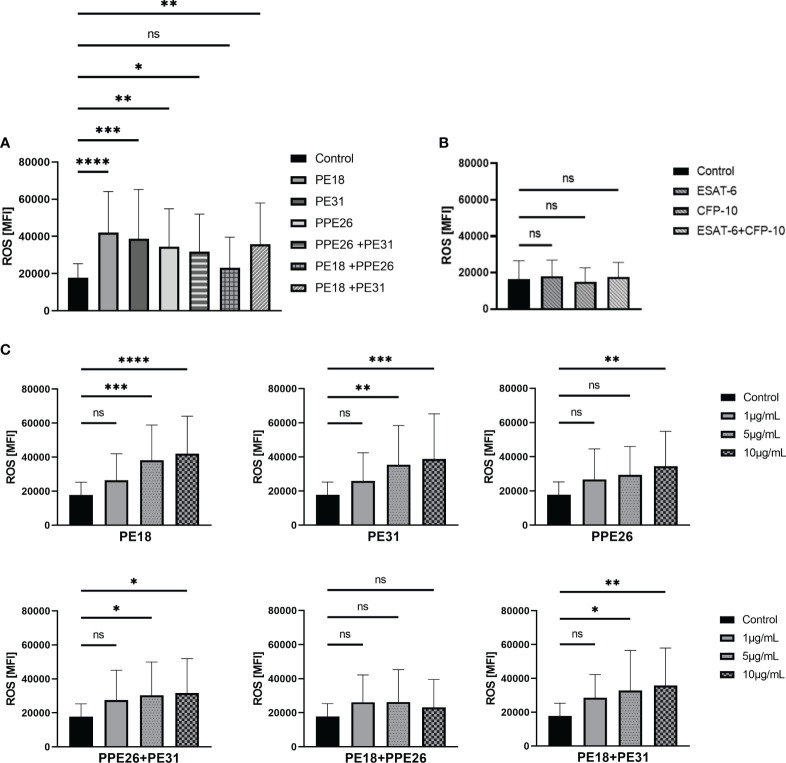
PE18, PE31, and PPE26 enhance ROS production in human neutrophils. **(A, B)** ROS production was assessed as mean fluorescence intensity (MFI) of intracellular H2DCF-DA after 3h incubation with different protein combinations at a concentration of 10µg/mL. **(A)** All PE/PPE candidates alone as well as the different combinations induced increased ROS production compared to **(B)** the negative control proteins ESAT-6 and CFP-10. **(C)** A concentration-dependent effect was also observed. The data presented in **(A, C)** were derived from the same experiments, utilizing the same control. Consequently, all data were collectively analyzed using a single one-way ANOVA test followed by Dunnett correction, compared to a common control (ns p>0.05, *p < 0.05, **p < 0.01, ***p < 0.001, ****p < 0.0001), and are depicted with mean ± SD. The data shown in **(B)** originates from separate experiments, and the statistical analysis involved employing a one-way ANOVA test with Dunnett correction compared to the control also represented with mean ± SD (all 3h incubation, n = 4).

First, we conducted screening assays with PMA-prestimulated neutrophils to adjust the experimental settings and to start a pre-screening with activated neutrophils. Here, the levels of ROS produced by neutrophils were quantified over an 80-minute time course after following priming with PMA and subsequent stimulation with the respective PE/PPE proteins, either alone or in combinations. The graphs show that PE/PPEs boost the generation of ROS after PMA stimulation compared to PMA alone (**Figure S3**). Detailed quantification of ROS at 3h after stimulation by flow cytometry is shown in **Supplementary Figure 4** including statistical evaluations (**Figure S4**). Then, the ability of PE/PPEs to stimulate ROS in untreated neutrophils was quantified. As depicted in **Figure 3**, the stimulation of human neutrophils with PE/PPE proteins at a concentration of 10µg/mL resulted in a significant induction of ROS. Specifically, this effect was observed when the proteins were administered individually, as well as in the combinations of PPE26+PE31 and PE18+PE31. Interestingly, the combination of PE18+PPE26, while not reaching statistical significance, exhibited an increase in ROS production compared to the medium control (**Figure 3A**). A concentration-dependent effect was also observed (**Figure 3C**). Therefore, the concentration showing the highest effect (10µg/mL) was selected to conduct further NET-induction experiments. Intracellular production of ROS was also assessed by *Mtb* proteins ESAT-6 and CFP-10. The two control proteins did not increase ROS at a concentration of 10µg/mL compared to unstimulated cells (**Figure 3B**).

### 3.4 Human neutrophils stimulated with PE/PPE candidates lead to NET release

**Figure 4:**
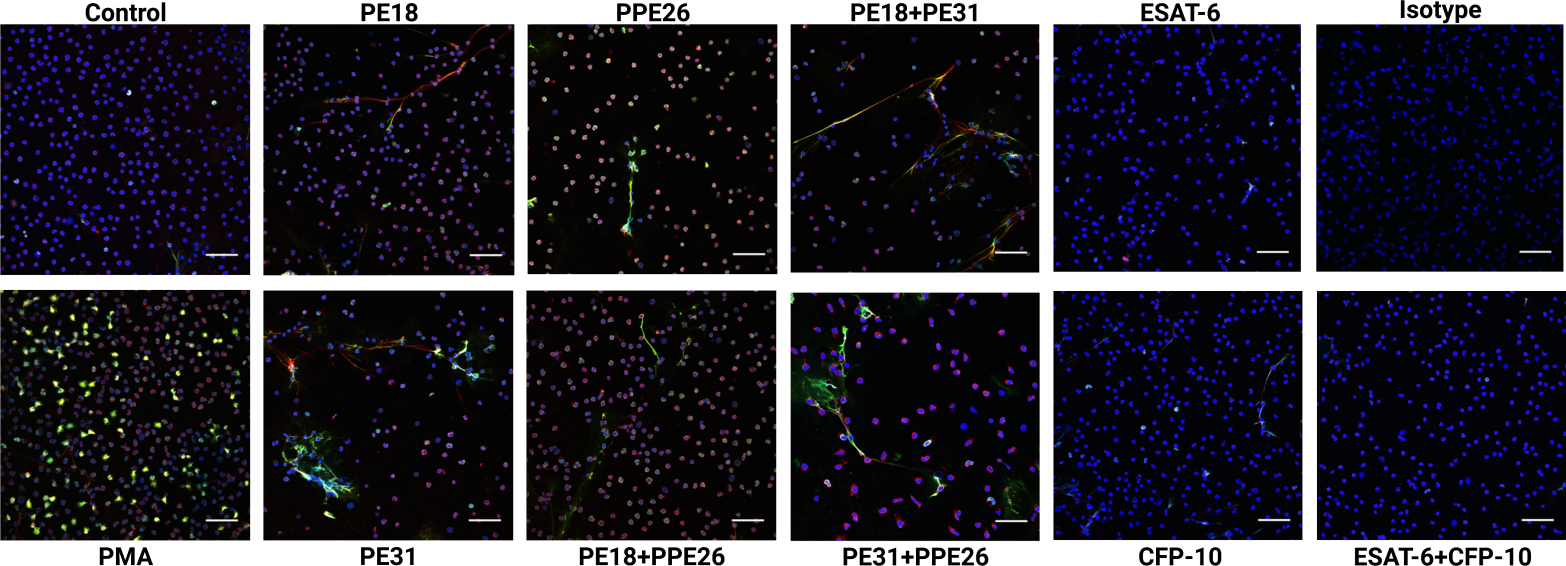
Neutrophils derived from healthy subjects were incubated with PE18, PE31, and PPE26 (10μg/mL) respectively for 3h. After staining, the cells were fixed and photographed by confocal microscopy. All settings were adjusted to respective isotype controls. Representative immunofluorescence images of NET-induction assays from different donors are shown. Staining: Blue= DNA; green= DNA/histone-1-complexes; red= myeloperoxidase. Scale bars, 50μm.

**Figure 5:**
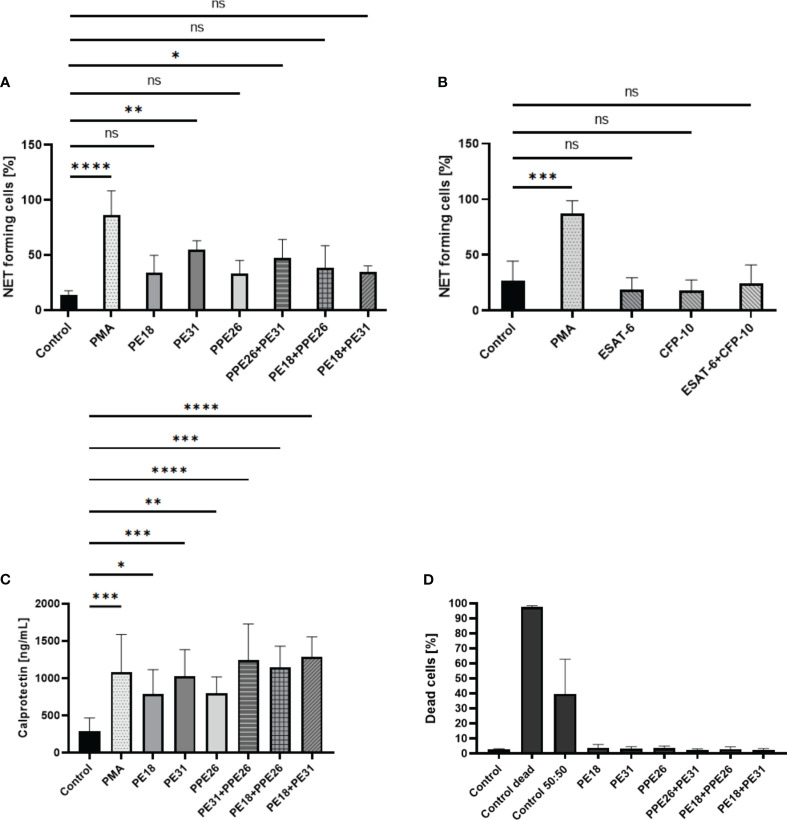
Statistical analysis of NET induction assays (10µg/mL, 3h stimulation) sorted by stimulus. **(A)** The percentage of NET-releasing cells is higher when cells are stimulated with PE/PPE candidates as single proteins but also with the different combinations, being significantly increased in the presence of PE31 individually, as well as the combination of this candidate with PPE26, compared to medium control. **(B)** Control proteins did not induce the formation of NETs. **(C)** Levels of calprotectin measured in supernatants collected after 3h stimulation of cells with respective PE/PPE proteins correlate with levels of NET-forming structures, with PE31 giving the highest response among the individual proteins and the combination of PE31 with both PPE26 or PE18 among the complexes. The data are presented as mean ± SD of three individual experiments and were analyzed with one-way ANOVA followed by Dunnett’s multiple comparison test. Per sample, six pictures were randomly taken from two slides and the number of NET-activated cells were determined from the total number of cells present in the slides. Unstimulated, PMA, PE/PPEs, and ESAT-6/CFP-10 from 3 independent experiments. **(D)** PE18, PE31, and PPE26 do not induce cell death in human neutrophils. Cell death was measured by PI staining in flow cytometry 3h post-infection with PE/PPEs. Every stimulus represents means (± SD) of four independent experiments. Heat-killed cells were used as a positive control (ns p> 0.05, *p < 0.05, **p < 0.01, ***p < 0.001, ****p < 0.0001).

Based on the screening results, we conducted the NET visualization and quantification analysis by confocal immunofluorescence microscopy after 3h incubation of the neutrophils with 10µg/mL concentration of the proteins as individual proteins and with the three different combinations. NETs were visualized by using different NET-specific markers being a combination of DAPI as cellular counterstain (53), MPO (53, 54), and DNA/histone-1-complex (55, 56). Here, we included three individual experiments (n=3 donors). From each sample/stimulus we randomly took six pictures from two slides per stimulus, and the number of NET-activated cells was determined from the total number of cells present in the slides. Our results indicate that all proteins and combinations have the potential to promote NET release as shown by a tendency in which all samples gave higher signals compared to control (**Figures 4**, **5A**). However, only PE31 and the combination of PE31+PPE26 seem to induce the effect of NET formation in a significant manner (**Figure 5A**). This effect was also seen when stimulating the cells with a much lower concentration of 1µg/mL of the respective individual PE/PPE proteins (**Figure S2**). Again, control proteins ESAT-6 and CFP-10 gave comparable levels of NET-forming cells to medium control (**Figure 5B**). Overall, we identified that the formation of PE/PPE-induced NETs seems to be a potential specific effect for these proteins, as no NETs were observed when neutrophils were stimulated with ESAT-6/CFP-10 *Mtb* proteins. Remarkably, PE31 appeared to have a more pronounced effect on NET formation (**Figure 5**).

### 3.5 PE/PPE candidates increase levels of calprotectin in human neutrophils

Neutrophils have been previously described to release calprotectin by forming NETs (57). Calprotectin is an antimicrobial cytosolic protein complex made up of proteins S100A8 and S100A9 involved in host defense. S100A8/A9 heterodimer is a major component of NETs and shown to accumulate in TB-induced granulomas (58). We analyzed levels of calprotectin in supernatants from human neutrophils stimulated with all three PE/PPE. As shown in **Figure 5C**, all candidates alone and with the different combinations induced significantly higher levels of S100A8/A9 after 3h stimulation compared to the negative control. Interestingly, PE31 and combinations that include this last candidate induced comparable levels to PMA positive control, similar to what we find in the NET-induction experiments. This phenotype was additionally confirmed when calculating the relative difference in the measured levels of calprotectin of PE/PPE-stimulated cells compared to the baseline control sample, with PE31 being the only protein doing so in a significant manner **(** **Figure S5-A** **)**. Once more, control proteins did not impact the calprotectin levels in human neutrophils **(** **Figure S5-B** **)**.

### 3.6 PE/PPE-stimulated human neutrophils release vesicular NETs

After the identification of NET structures released from human neutrophils stimulated with the three PE/PPE candidates, we wanted to further investigate the process behind the formation of NETs. It is important to mention that most plasma cell membranes seem to be pretty much conserved upon release of NETs (**Figure 4**). Therefore, PE/PPE-induced NETs may be released in vesicles without the disruption of the plasma membrane allowing the cells to conduct further biological functions. To investigate that, we first assessed whether all three PE/PPE candidates would lead to cell death by determining the mean fluorescence intensity (MFI) of intracellular PI in stimulated neutrophils. As shown in **Figure 5D**, none of the candidates alone or with the different combinations promote cell death. To assess that, we applied transmission electron microscopy (TEM) to PE31-stimulated neutrophils to further discriminate the status of the cell structure and membranes from granules, nucleus, and plasma membrane. Additionally, we were interested in analyzing the localization of PE31, our most promising candidate, as shown by its implication in ROS production and NET-formation in the present study.

**Figure 6:**
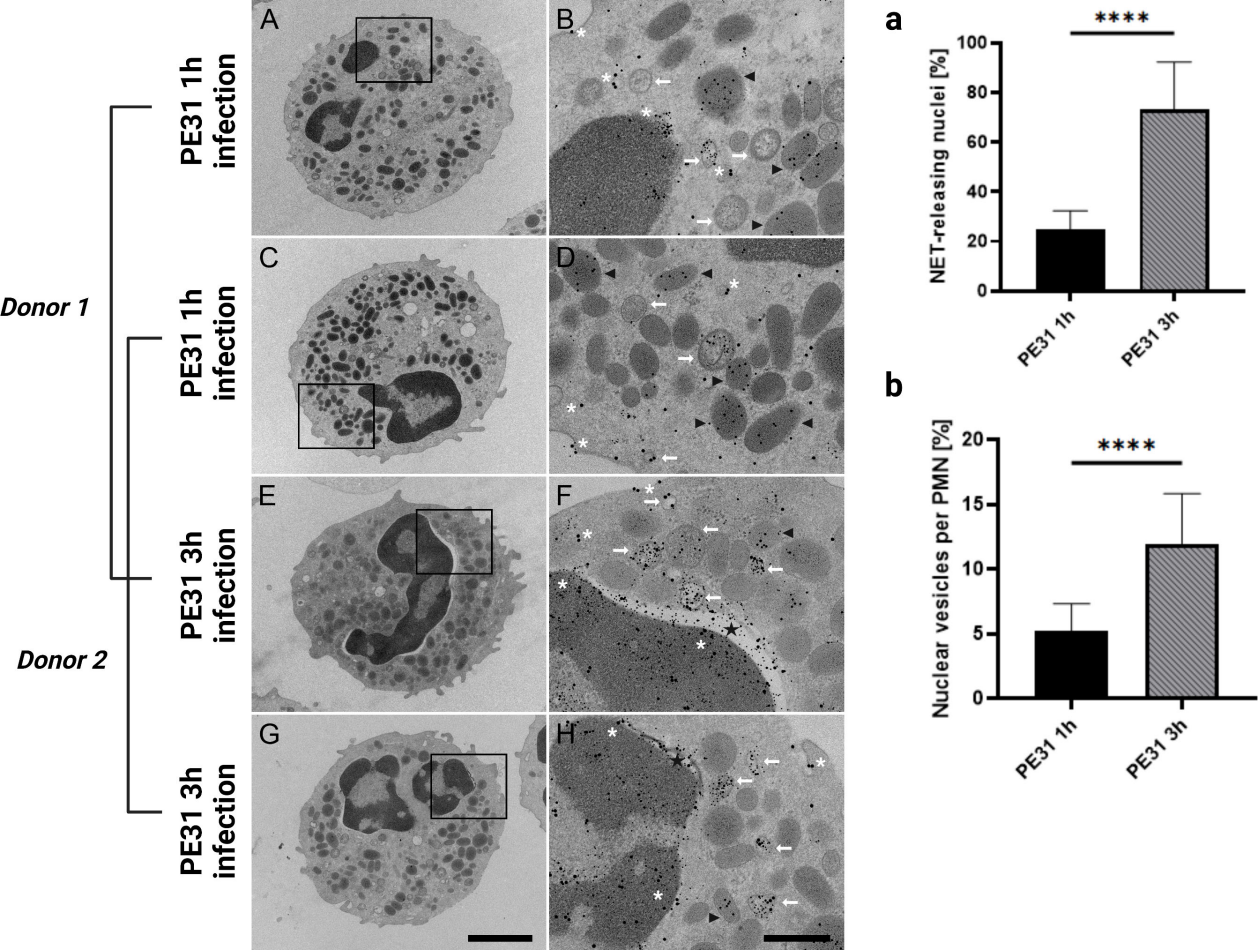
Transmission electron microscopy of NET formation showing nuclear envelope alterations and nuclear vesicle formation. Neutrophils were treated as described before, and after fixation with glutaraldehyde, they were processed and studied with transmission electron microscopy. Images were obtained from samples from two different donors with neutrophils incubated with antigen PE31 for 1h or 3h. Zoom-in images show neutrophils with DNA-containing vesicles in the cytoplasm that are positive for NE and H3cit (white arrow). The nuclear envelope of PE31-stimulated neutrophils appears intact in earlier and later infection times. **(A-D)** After 1h incubation, PE31 can be found included in NE positive organelles (black arrowhead) as well as free in the cytoplasm or binding to the plasma membrane (white asterisk). In a later stage of exposition time at 3h **(E-H)**, antigen PE31 is additionally included in nuclear or NET vesicles (white arrow) containing a mixture of the antigen with the NE and H3cit NET-markers and is also seen binding to the nuclear membrane. Furthermore, nuclear vesicle formation seems to increase over time **(E-H)**. Importantly, these nuclei contain nuclear envelope dilatations (black star) referred to as the separation of the inner nuclear membrane from the outer nuclear membrane **(E-H)**. Statistical analysis of the TEM images from one single donor comprise 30 cellular profiles from randomly selected fields on the thin sections that were analyzed per sample **(a, b)**. Neutrophils stimulated with PE31 for 3h showed a significantly higher percentage of cells containing vesicular-NET releasing nuclei **(a)** as well as higher counts of nuclear vesicles (“vital NETosis”) compared to 1h stimulated cells **(b)**. Data were analyzed with the paired t-test (n= 30) and presented with mean ± SD (****p < 0.0001). Sections were stained with uranyl acetate and lead citrate (see Materials and Methods), where 5 nm gold labeling= H3-cit; 10 nm gold labeling= NE; 15 nm gold labeling= PE31. Scale bars in cell overview pictures= 2μm; zoom pictures= 250nm.

**Figure 7:**
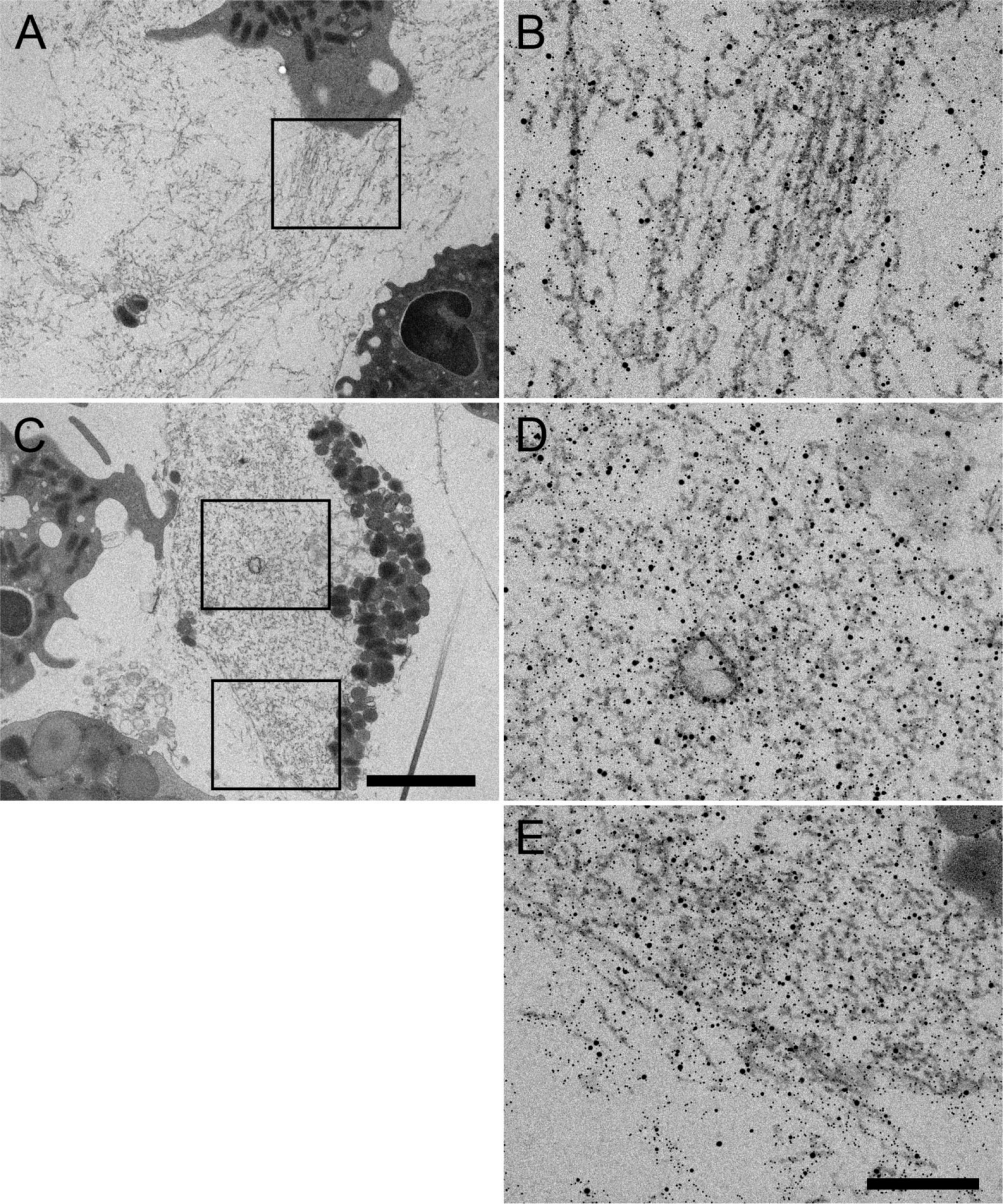
Representative TEM images of neutrophils at 3h post-stimulation with PE31 showing cytoplasmic and extracellular NETs. **(A)** Overview of extracellular NETs with respective zoom-in image **(B)** with NETs shown as fibrillary structures with NE, H3cit, and PE31 bound. **(C)** Example micrograph containing both cytoplasmic and extracellular NETs where **(D)** shows the zoom-in/immunolabeling of cytoplasmic NETs, and **(E)** shows the respective zoom-in/immunolabeling of a mixture of cytoplasmic and extracellular NETs with PE31 bounding to these structures that are double positive for NE and H3cit. Sections were stained with uranyl acetate and lead citrate (see Materials and Methods), where 5 nm gold labeling= H3-cit; 10 nm gold labeling= NE; 15 nm gold labeling= PE31. Scale bars in cell overview pictures= 2μm; zoom pictures= 250nm.

Therefore, we analyzed the neutrophils from two different donors with TEM after 1h and 3h infection for NET formation by immune gold-labeling of antigen PE31, NE, and H3cit (**Figure 6**). As shown in **Figure 6**, PE31 stimulation induced enhanced formation of DNA-containing vesicles (H3cit- and NE-positive vesicles) as identified inside neutrophils by immune gold-labeling in antigen-stimulated neutrophils compared to that from the negative control (no antigen), in which they are rather occasional **(** **Figure S6** **)**. Whereas in the case of the control the nuclear envelop appears intact and with no apparent modifications, PE31-stimulated neutrophils showed dilatation between the inner and the outer nuclear membrane after 3h (**Figures 6E–H**), indicating that the nuclear membrane is furthermore damaged after long-time exposure, as also shown as vital NET-release by Pilsczek et al. (15). Interestingly, PE31 binds to both the nuclear membrane and the plasmatic membrane, but it accumulates especially in the nucleus and nuclear vesicles. Furthermore, significant differences occur when neutrophils are triggered by antigen PE31 for 3h compared to the 1h time point, leading to a higher number of neutrophils that are releasing vesicular NETs (**Figure 6a**) as well as an increased number of NET-vesicles released by neutrophil’s nucleus in the 3h samples (**Figure 6b**). Additionally, detailed scanning electron micrographs confirm the presence of fibrinous NET structures (DNA strands containing bound H3cit, NE, and PE31) present in the cytoplasm and in the extracellular space of neutrophils stimulated with PE31 (**Figure 7**). All together, these results indicate that PE31 stimulates human neutrophils to form NETs, but these cells remain intact and show a phenotype of vesicular NET release.

## 4 Discussion

In the present study, we characterized PE/PPE antigens from *Mtb* PE18, PE31, and PPE26, in human-derived blood neutrophils by showing the production of ROS and subsequent NET release at 3h post-stimulation. Moreover, PE/PPE-stimulated neutrophils remained intact with no cell death rather suggesting a phenotype of vital NET formation. Thus, our data show that PE/PPE proteins play a central role in the host-pathogen interaction of innate immune responses.

PE/PPE proteins are relevant immunogens (59), especially those included in the ESX5 gene cluster (25), and some of them are known to directly interact with cells from the host immune system. Thus, PE18 can be localized in the cell wall or secreted (60). Similarly, PE31 plays a role in transforming the surface properties of *Mycobacterium smegmatis* (26), and antigen PPE26 interacts directly with the host via TLR2 in murine macrophages (34). Together, this suggests a potential direct interaction of these proteins with the host. Notably, PE/PPEs can be organized in complexes, which may further increase their functional diversity and complexity, and contribute to antigen presentation (31, 32), enabling them to elicit and modulate host immune responses (61). Interestingly, here we provide insights into the complex formation for the PE/PPE family of proteins by studying protein-protein interactions between PE18 and PPE26, as well as PE31, an ESX5-associated protein with high homology with PE18, in complex with the same PPE26 protein, assayed by biolayer interferometry. Binding of PE18 to PPE26 was robust, yielding a dissociation constant (K_D_) of 0.61 ± 0.032µM while K_D_ value for PE31-PPE26 was slightly decreased (2.70 ± 0.62µM) (**Figure 2D**). In contrast, binding of PE18 and PE31 to negative control (unloaded sensor) was undetectable. Therefore, our bioinformatics and experimental data support previous studies where PE18 and PPE26 were predicted to form a pair (48), and add new insights into other possible combinations for complex formation as it is the case of PE31 with PPE26.

During infection, host cells promote the generation of ROS as a defensive mechanism (62). Importantly, augmented neutrophil counts in ATB patients as well as elevated levels of different NET-markers in plasma samples from TB subjects that correlate with disease severity, suggest that NETs may play an important role in TB immunopathology (10, 63). Thus, strategies that target neutrophil treatment may be beneficial.

NET formation is an antimicrobial mechanism by neutrophils in which a backbone of DNA decorated with histones and different antimicrobial granular proteins including the enzymes NE and MPO, is released by these innate immune cells in an attempt to trap or kill the pathogenic agent (4). In contrast, excessive release of extracellular NETs can lead to inflammation and tissue damage (64, 65). During infection, neutrophils react to *Mtb* by performing different antimicrobial mechanisms such as degranulation, phagocytosis, necrosis, or production of ROS, thus directly or indirectly activating other immune cells and contributing to mycobacterial clearance. However, *Mtb* has also developed strategies to survive the host immune system. Importantly, *Mtb* can activate neutrophils to form NETs (5, 66). This phenomenon occurs *in vitro* and *in vivo*. Thus, *Mtb*-induced NETs have been detected in sputum (11), lung tissue, and peripheral blood of TB patients (10), further supporting the relevance of this response during *Mtb* infection. Similarly, NET formation has also been characterized upon stimulation with the classical *Mtb* ESAT-6 protein (an ESX1-derived antigen) resulting in controversial findings by two different studies. On one hand, neutrophils were found to undergo nuclear changes when stimulated with ESAT-6 and CFP-10 *in vitro*, but no NET structures were identified (50). On the other hand, a different study showed the formation of NETs by ESAT-6-treated necrotic human-derived neutrophils as referred to by co-localization of ESAT-6 and MPO (19). MPO translocated to the nucleus is a specific marker for NETs, the same as NE and citrullinated histones (H3Cit) (53, 54, 67). Nevertheless, immunostaining or assessment of MPO levels by flow cytometry alone may not indicate the formation of NETs on its own, as shown by previous work where reviewing existing methodologies for the visualization and quantification of NETs (40, 68). Furthermore, the simple presence of mixed cell-free DNA and granular protein MPO in the external environment of the cells is not considered specific for NET formation, since it can also be associated with other cell death processes like necrosis. Therefore, additional investigations are necessary to clarify the precise function of ESX1-secreted proteins in the formation of NETs and their impact on the overall functionality during *Mtb* infection. Importantly, more complete setups or techniques, such as immunofluorescence microscopy through staining of combined specific NET-markers, may help in the recognition of NET structures (53, 69, 70). In the present study, we employed immunofluorescence microscopy to visualize and quantify NETs by utilizing specific markers for neutrophils and NETs. Our findings revealed no significant induction of NETs by ESAT-6 and CFP-10 *in vitro*, which aligns with the observations reported by Rojas-Espinosa et al. (50).

The mechanisms by which PE/PPEs contribute to the interaction of the mycobacteria with the host remain unknown. Even so, diverse functions have been attributed to this unique family of proteins, including modulation of host immune responses and manipulation of host cell fates. PE/PPEs modulate different forms of cell death such as apoptosis (26, 71–73), necrosis (74–76), pyroptosis (77) and autophagy (78). However, to our knowledge, there was no study published until now that would discuss the role of PE/PPEs in the context of NET formation.

In this study, we observed for the first time that PE18, PPE26, and with the highest impact, PE31, as well as their combinations, induced the formation of NETs in human neutrophils after 3h stimulation *in vitro*, in correlation with enhanced production of ROS, what appeared to be dependent on the concentration of protein used. Thus, PE/PPE-induced NET structures might be formed by a similar mechanism to PMA-stimulated NETs (79). However, in contrast to PMA-induced NETosis, PE/PPE proteins did not induce cell death as determined by levels of PI measured by flow cytometry and the appearance of the structure and plasma membrane of most cells, which seemed pretty much conserved as observed by confocal microscopy. These findings suggest that PE/PPEs may rather lead to the formation of vesicular or vital NETs. In addition to the well-characterized mechanism of “suicidal” NETosis, which involves the release of extracellular DNA fibers into the extracellular space resulting in cell death, there is an alternative “vital” mechanism for NET release. In this process, vesicles containing NET components are transported into the cytoplasm, and their contents can be released into the surrounding milieu while maintaining the integrity of the plasma membrane (17). The release of vital NET structures can be triggered by ROS-dependent or ROS-independent mechanisms (17, 18). In the present study, levels of intracellular ROS were assessed by loading cells with H2DCF-DA and analyzed by flow cytometry. Whereas all three individual proteins showed significant production of ROS after stimulation of neutrophils compared to medium control, ESAT-6 and CFP-10 control proteins induced ROS levels similar to negative control after 3h stimulation. Furthermore, PE/PPEs boosted levels of ROS in PMA-primed neutrophils. This indicates that PPE26, PE18, and PE31 can trigger intracellular signaling pathways that lead to the generation of ROS in neutrophils highlighting the pro-inflammatory and antimicrobial properties of this unique family of proteins. Additionally, we used immunofluorescence confocal microscopy to observe extracellular NET structures by staining NET-specific markers: extracellular DNA, MPO, and the DNA-histone-1-complex, demonstrating that PPE26, PE18, and PE31 produced ETs in neutrophils *in vitro*. Nevertheless, significances were only found upon stimulation with PE31 and one of the combinations (PPE26+PE31). These results were supported by increased levels of calprotectin (S100A8/A9 heterodimer), a major component of NETs showed to accumulate in TB-induced granulomas (58, 80), as measured by ELISA in supernatants of samples stimulated with the three proteins and the different combinations. Notably, PE31 seems to potentiate the effect of PE18 in the secretion of calprotectin, as shown by an increase in the levels of calprotectin that may be bound to NETs. This may be due to a synergistic effect when the two proteins are combined that translates into potentiated signaling pathways or transcriptional regulators responsible for calprotectin expression. Furthermore, this tendency is also observed when combining PE31 and PPE26 **(** **Figure 5C**). Importantly, there is evidence of complex formation for PE proteins, as it is the case for PE9 (Rv1088) and PE10 (Rv1089) that have been shown to form a pair that interacts with THP-1 macrophages via TLR4 signaling, resulting in apoptosis and modulation of cytokine levels (73). Thus, the combination of PE proteins might in some cases exhibit an additive or potentiated impact on the modulation of neutrophil function.

Whereas ESAT-6 was suggested to induce the formation of NETs (19), in this study ETs were not observed upon stimulation of neutrophils with mycobacterial antigens ESAT-6 and CFP-10, in correlation with the absent production of ROS by these two proteins. Furthermore, after applying transmission electron microscopy we found that PE31 leads to activation of vesicular NET formation and that it can bind to membrane structures in neutrophils. Thus, PE31 may bind directly to surface receptors in neutrophils since *pe/ppe* gene clusters encode proteins that are surface exposed as well as secreted proteins that can interact directly with their host targets via TLR2/4 (34, 73, 81), affecting host-pathogen interactions and ultimately activating anti-inflammatory or pro-inflammatory responses. Therefore, this effect could also be extended to other PE/PPE proteins. Finally, the formation of NETs induced by *Mtb* PE/PPEs may remove the anti-mycobacterial threat of the neutrophil while simultaneously contributing to mycobacterial clearance and triggering the innate immune response, as for example, previous studies have shown that NETs can directly interact with macrophages through phagocytosis resulting in the activation of these cells during the early stages of *Mtb* infection (66). Furthermore, this can enhance the immune response against *Mtb* and promote the initiation of adaptive immunity. Thus, the formation of NETs by PE18, PE31, and PPE26 may have significant implications for the development of future host-directed therapies for TB. However, further investigations are required to determine if this phenomenon benefits or harms the host. Comparatively, the impact of NETs on other bacteria is relatively better understood. For example, when NETs are found in the non-lytic form, these structures seem to be beneficial by reducing bacterial burden upon infection with *Staphylococcus aureus* (15). However, an excessive release of NETs may lead to detrimental consequences for the host, since cytotoxic NET-associated molecules can cause inflammation and tissue injury. In this regard, abnormal NET formation or impaired NET degradation has been associated with autoimmune diseases like Anti Neutrophil Cytoplasmic Antibody (ANCA) associated vasculitis (AAV) (82).

In conclusion, we report a novel and specific function for the PE/PPE family of proteins consisting of the formation of NETs and their potential use in host-directed strategies against TB. Release of NET structures correlated with increased production of ROS by human neutrophils stimulated with the three proteins. However, PE/PPEs did not induce cell death pointing towards a vital form of NET formation that would allow cells to perform different antimicrobial mechanisms helping in fighting the infection. These findings provide evidence that PPE26, PE18, and PE31 can regulate neutrophil functions and actively contribute to immune responses linked to the formation of NETs. Furthermore, these results warrant further investigation regarding the characterization of these mycobacterial proteins as immunodominant antigens, an area currently under active exploration by the authors.

## Data availability statement

The original contributions presented in the study are included in the article/**Supplementary Material.** Further inquiries can be directed to the corresponding author.

## Ethics statement

The studies involving humans were approved by Ethical committee of the Hannover Medical School Nr. 3295-2016. The studies were conducted in accordance with the local legislation and institutional requirements. The participants provided their written informed consent to participate in this study.

## Author contributions

MG-B, MMe, MSt, WO, and MMö performed the experiments. MG-B, MMe, MSt, WO, AE, MSi, MMö, and MK-B analyzed the data. MG-B, MMe, MSt, WO, AE, MSi, MMö, and MK-B designed the experiments. MMe, MSt, WO, AE, MSi and MK-B supervised the experiments. MMe, MSi, and MK-B reviewed the manuscript. MG-B, MMe, and MK-B wrote the manuscript. All authors contributed to the article and approved the submitted version.
