# Supplementary material for: Mycobacterium tuberculosis PE/PPE proteins enhance the production of reactive oxygen species and formation of neutrophil extracellular traps
Source: Front Immunol. 2023 Aug 22;14:1206529. doi: 10.3389/fimmu.2023.1206529 (PMC10478095; doi:10.3389/fimmu.2023.1206529)
Supplement: Supplementary file 1 [file DataSheet_1.docx]

Supplementary Material

*Mycobacterium tuberculosis* PE/PPE proteins enhance the production of reactive oxygen species and formation of neutrophil extracellular traps

**María García-Bengoa^1,2,3^, Marita Meurer^1,2^,** **[Matthias Stehr](https://pubmed.ncbi.nlm.nih.gov/?term=Stehr+M&cauthor_id=22982567)^3^, [Ayssar A Elamin](https://pubmed.ncbi.nlm.nih.gov/?term=Elamin+AA&cauthor_id=29045498)^3^,** **Mahavir Singh^3^,** [**Wulf Oehlmann**](https://pubmed.ncbi.nlm.nih.gov/?term=Oehlmann+W&cauthor_id=21603219)**^4^, Matthias Mörgelin^5^, Maren von Köckritz-Blickwede^1,2^**

^1^Department of Biochemistry, University of Veterinary Medicine Hannover, Germany

^2^Research Center for Emerging Infections and Zoonosis (RIZ), University of Veterinary Medicine Hannover, Germany

^3^LIONEX Diagnostics and Therapeutics GmbH, Braunschweig, Germany

^4^UGA Biopharma GmbH, Hennigsdorf, Germany

^5^Colzyx AB, SE-223 63 Lund, Sweden

*** Correspondence:** Prof. Dr. Maren von Köckritz-Blickwede
mkoeckbl@tiho-hannover.de

# Supplementary Figures and Tables

## Supplementary Tables

| **Table S1. Primers used for adding 6xHis-tag to PE18 and PE31.** | |
| --- | --- |
| Gene | Sequence (5’-3’) |
| PE18-F | GCGCCGCCATGGATTCCTTTGTGACG |
| PE18-R | CGCGCGCAAGCTTAGTGATGGTGATGGTGATGTCCTGCTGCAGCCGCATTAG |
| PE31-F | GCGCCGCCATGGTCTCGTTTACCGCG |
| PE31-R | CGCCGCAAGCTTAGTGGTGATGATGGTGATGCCCGGTCACAACCGCGTTTG |

| **Table S2. Primers used for sequencing.** | |
| --- | --- |
| Vector | Sequence (5’-3’) |
| pWo1022-F | GAGCGGATAACAATTTCAC |
| pWo1022-R | AGGTGGCTAGCTGATCA |
| pET-28a-F | GATTATGCGGCCGTGTACAA |
| pET-28a-R | TTGTACACGGCCGCATAATC |
| pETDuet-1-F | AACCCCTCAAGACCCG |
| pETDuet-1-R | TCCCGCGAAATTAATACG |

| **Table S3. List of antibodies used in this study.** | |  |  |
| --- | --- | --- | --- |
| Name | Supplier | Catalog number | Concentration used |
| polyclonal mouse anti-His | Qiagen | 34660 | 1:100 |
| polyclonal rabbit anti-E.coli BL21 (DE3) | Dako/Agilent | B0357 | 1:167 |
| goat anti-mouse IgG-HRP | Thermo Scientific | 31432 | 1:5000 |
| goat anti-rabbit IgG-HRP | Pierce (Perbio) | 31462 | 1:5000 |
| mouse monoclonal IgG2a anti-DNA/histone | Millipore, Billerica, Massachusetts, USA | MAB3864 | 1:1000 |
| rabbit anti-human myeloperoxidase | Dako | A0398 | 1:360 |
| Alexa-Fluor-488-labelled goat-anti-mouse IgG2a | Invitrogen | A32723 | 1:1000 |
| Alexa-Fluor-633-labelled goat-anti-rabbit IgG | Thermo Scientific | A21070 | 1:1000 |
| Isotype control murine myeloma IgG2a | Sigma | M5409-1MG | 1:100 |
| Isotype control IgG from rabbit serum | Sigma | I5006 | 1:60 |
| gold-labeled Anti-Histone H3 (citrulline R2 + R8 + R17) | Abcam, Berlin, Germany | ab5103 | 1:80 |
| anti-neutrophil elastase | Abcam, Berlin, Germany | ab131260 | 1:80 |
| gold-labeled anti-PE31 polyclonal rabbit antibody | LIONEX GmbH, Braunschweig, Germany | - | 1:50 |

## Supplementary Figures

**
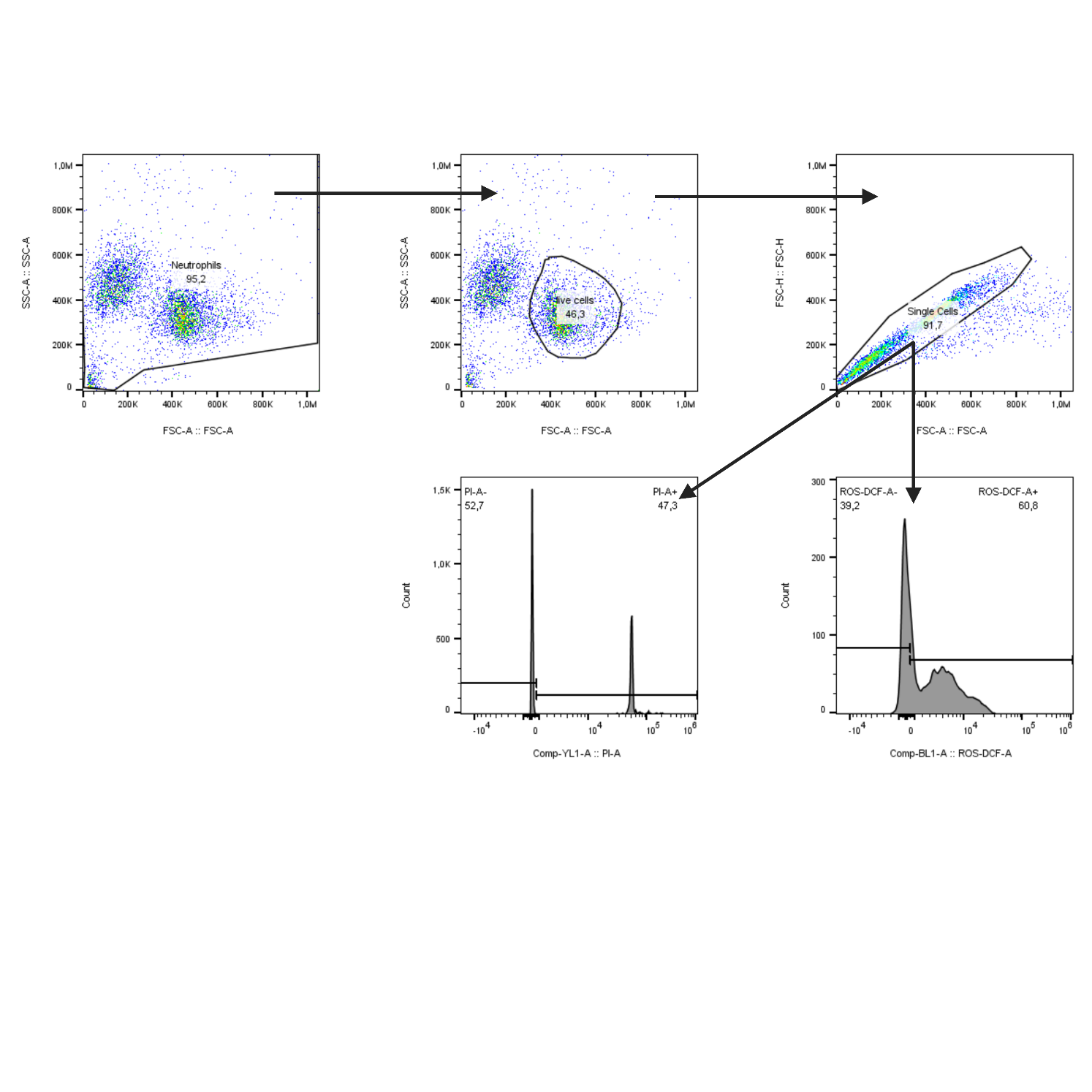
**

**Supplementary Figure 1.** Sequential gating to identify neutrophils producing intracellular ROS. A mixed population of viable and dead cells is represented in this figure. First, it appears a representative side scatter (SSC) versus forward scatter (FSC) plot showing all cells collected from the sample. Then, neutrophils were gated based on their characteristic SSC and FSC properties and distinct population clustering. Subsequent gating on single cells is performed to exclude cell aggregates and debris. Within the single neutrophil population, gating ROS-producing neutrophils is performed using the H2DCF-DA dye that allows discrimination of this population from the non-ROS-producing cells. Similarly, dead cells are excluded based on staining with PI. In order to distinguish between living and dead cells, gating parameters were established based on the dead control sample. Consistent gating thresholds were applied to all flow cytometry analyses within a single experimental setup. Acquisition volume was set to 50µL, and all cells in this volume were counted. Acquisition speed was set to 100µL/min.

**
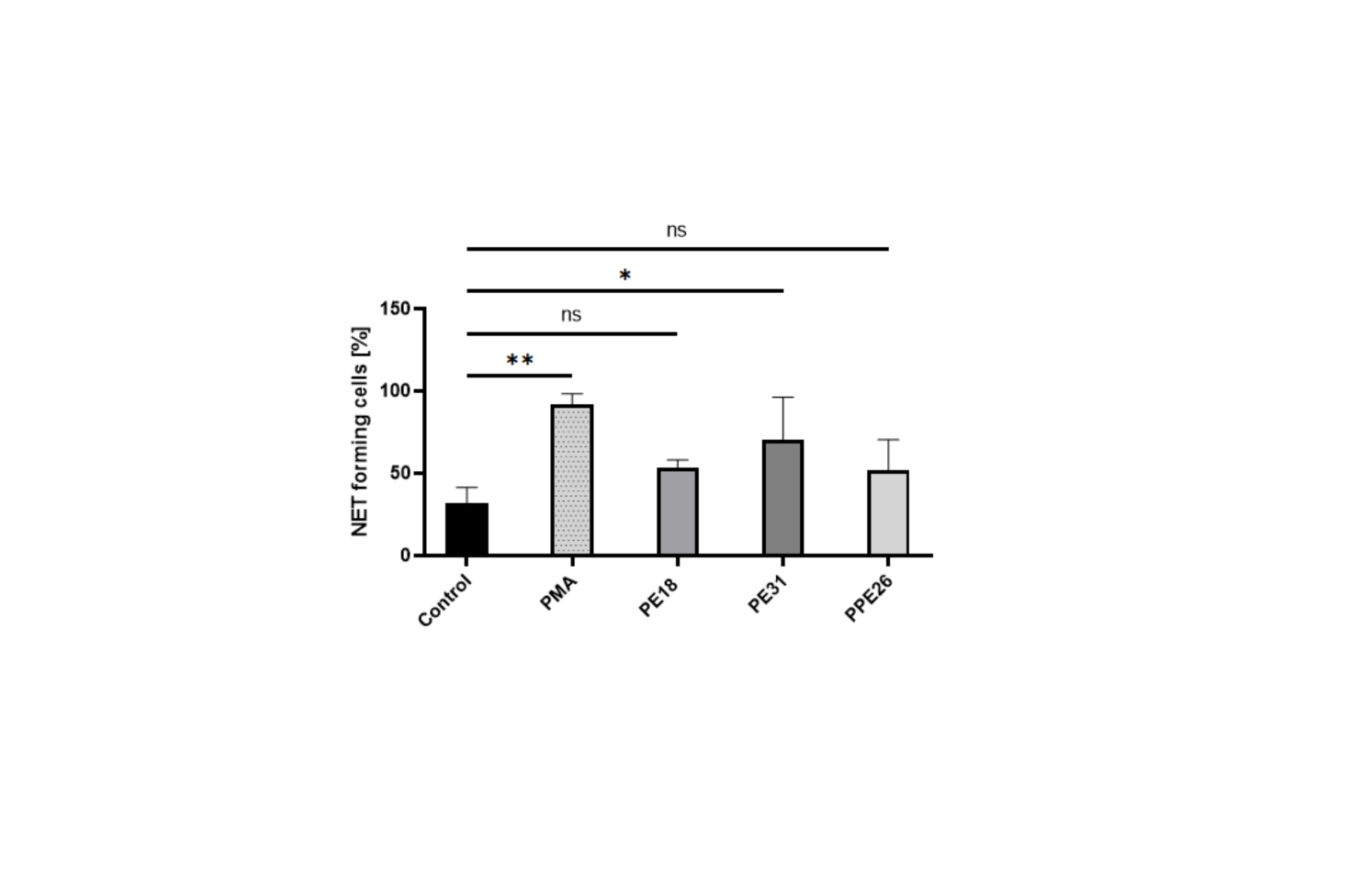
**

**Supplementary Figure 2.** Statistical analysis of NET induction assays with the lowest concentration of 1µg/mL of the single PE/PPE proteins after 3h stimulation. Significant differences are found when cells are stimulated with candidate PE31 compared to the control. The data are presented as mean ± SD of three individual experiments from individual donors and were analyzed with one-way ANOVA followed by Dunnett’s multiple comparison test. Per sample, six pictures were randomly taken from two slides and the number of NET-activated cells were determined from the total number of cells present in the slides. Unstimulated, PMA and PE/PPEs from 3 independent experiments (*ns p> 0.05, * p < 0.05, ** p < 0.01*).


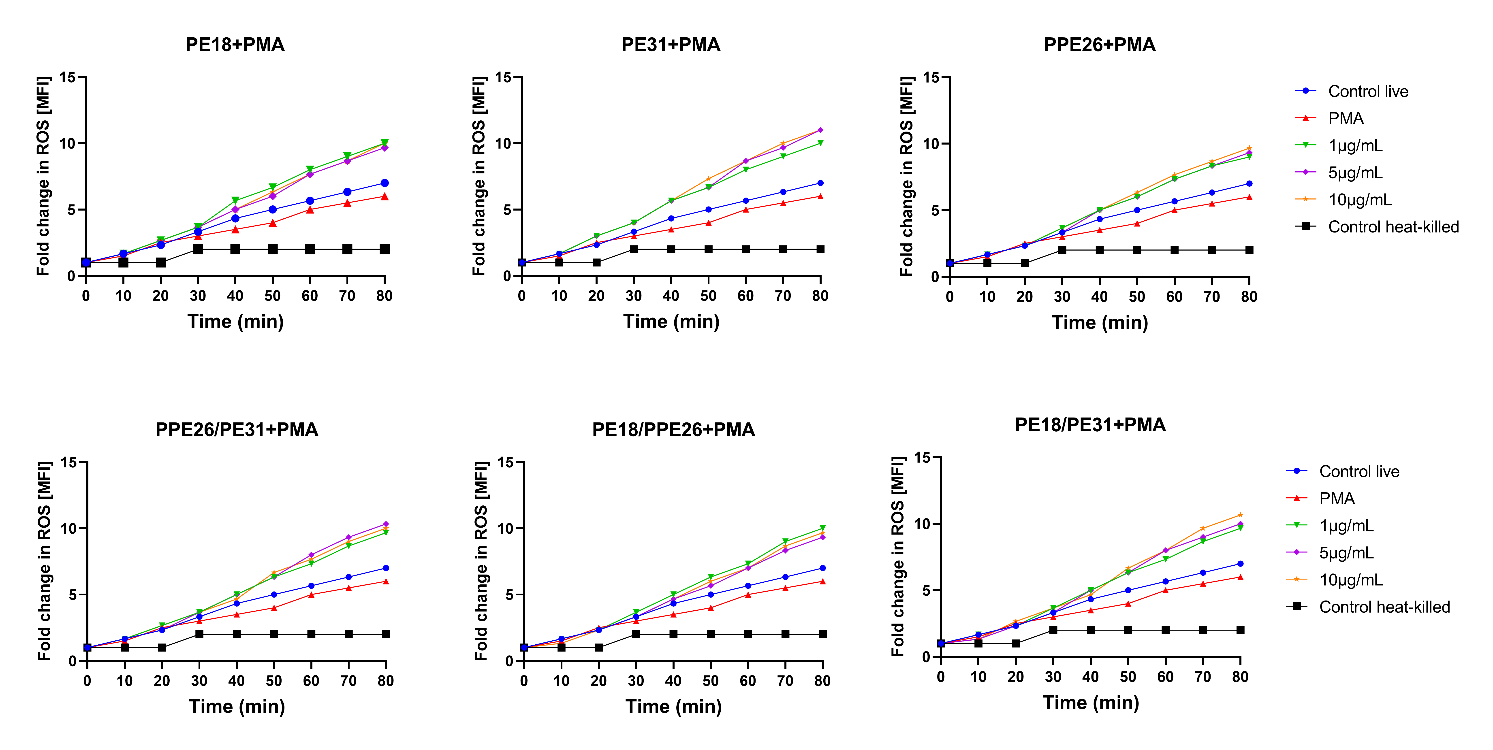


**Supplementary Figure 3.** Production of intracellular ROS induced by proteins PE18, PE31, and PPE26 is boosted in the presence of PMA. PMA (25nM) was used to pre-activate the neutrophils for ROS production**.** ROS production was assessed as mean fluorescence intensity (MFI) of intracellular H2DCF-DA over a period of 80 minutes by real-time measurement of ROS levels in stimulated cells by a Tecan fluorescence reader. Notably, neutrophils stimulated with PMA in combination with PE/PPE proteins exhibit distinctly higher levels of ROS compared to cells stimulated with PMA alone, as indicated by the diverging curves. All data are presented with mean ± SD (n= 4).

**
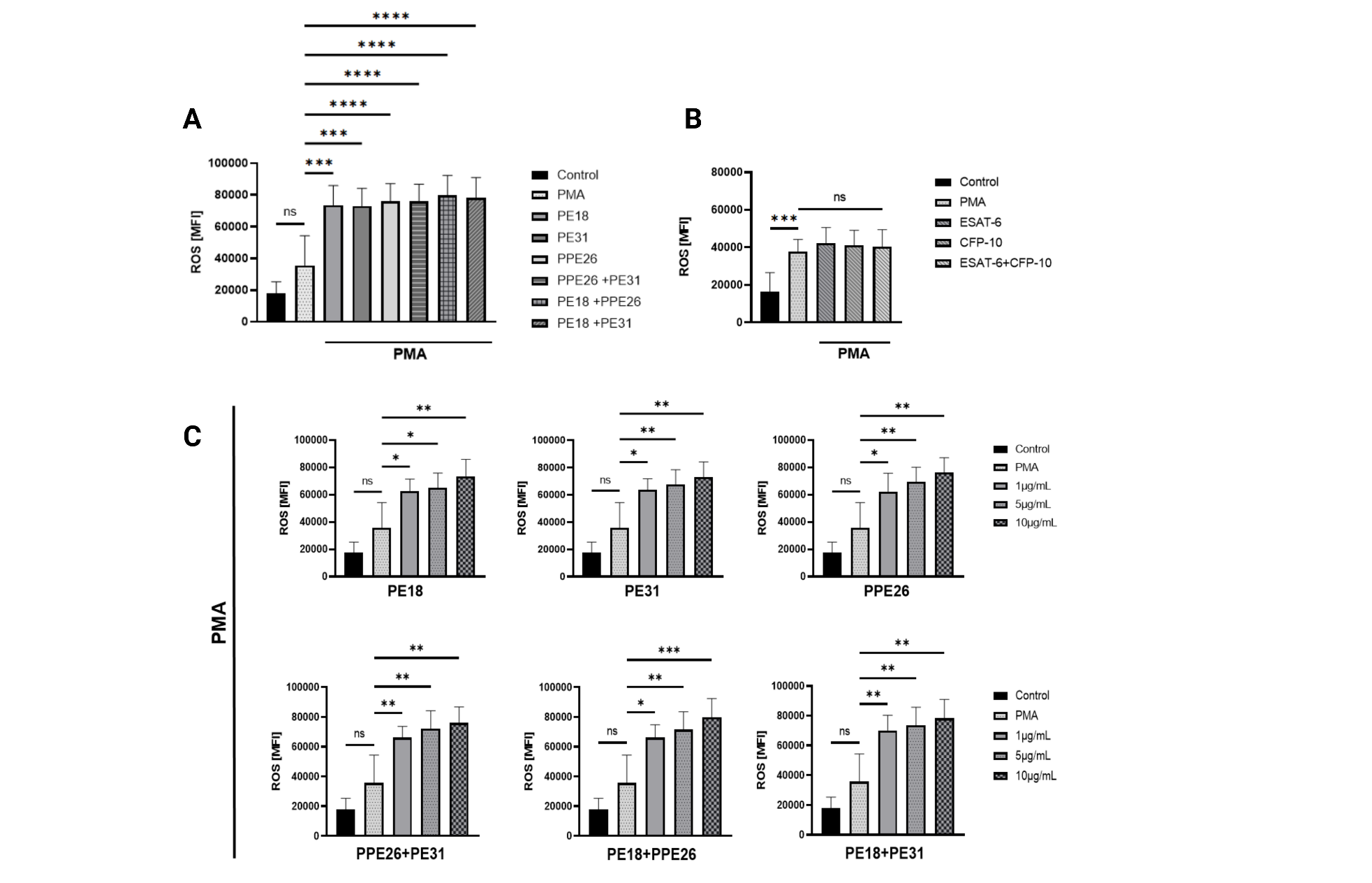
**

**Supplementary Figure 4.** Production of intracellular ROS induced by proteins PE18, PE31, and PPE26 is boosted in the presence of PMA. **(A-C)** ROS production was assessed as mean fluorescence intensity (MFI) of intracellular H2DCF-DA after 3h stimulation with different protein combinations and concentrations in the presence of PMA using flow cytometry. (**A-B**) All PE/PPE candidates alone and the combinations increased the production of intracellular ROS in neutrophils pre-stimulated with PMA compared to control proteins ESAT-6 and CFP-10. (**C**) A concentration-dependent effect was also observed after 3h stimulation. All data were analyzed with one-way ANOVA test followed by Dunnett correction (*ns p> 0.05, * p < 0.05, ** p < 0.01, *** p < 0.001, **** p < 0.0001*) and are presented with mean ± SD (n= 4).

**
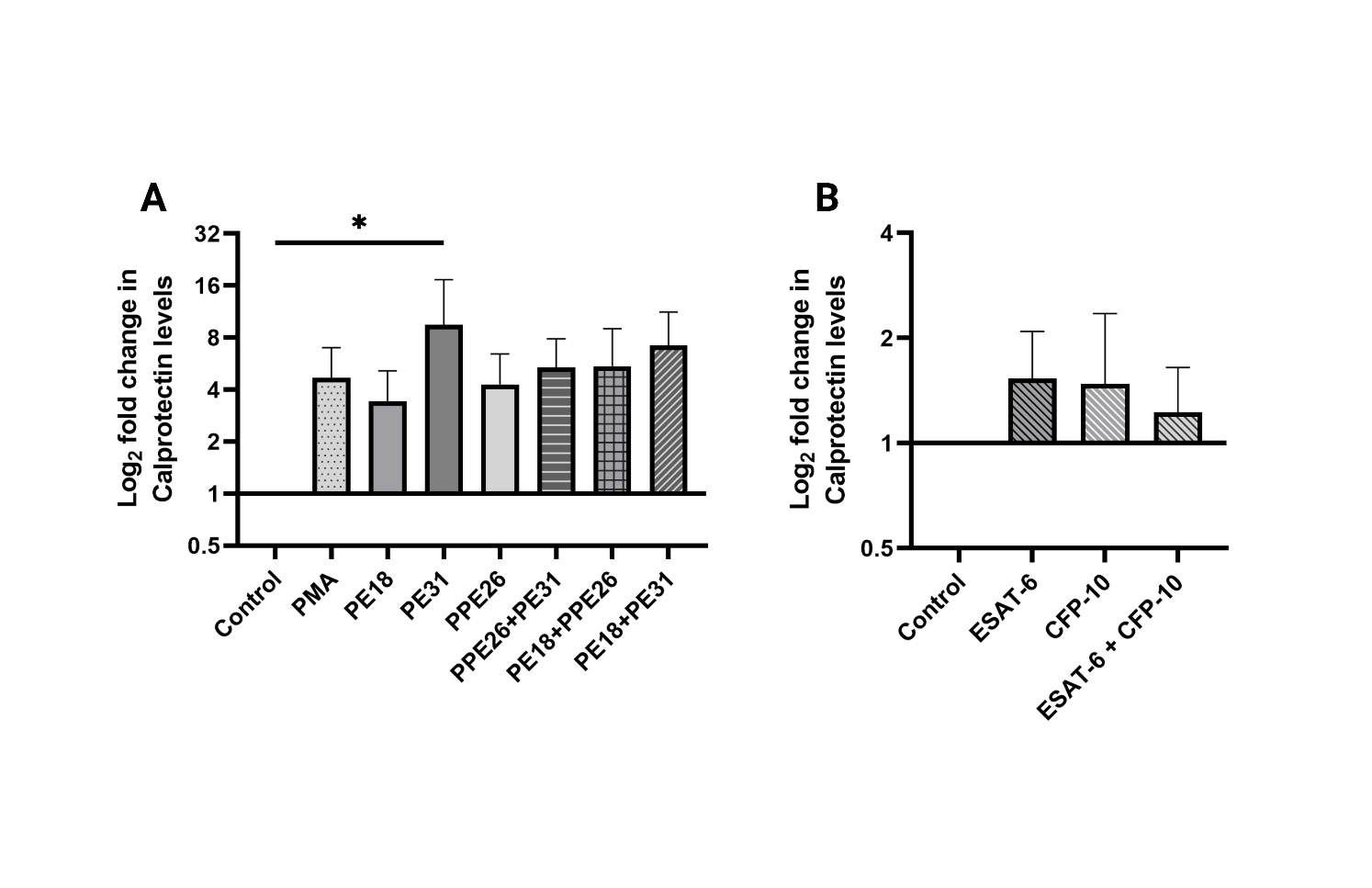
**

**Supplementary Figure 5.** Differences in the levels of calprotectin of PE/PPE stimulated human neutrophils relative to an unstimulated control sample. Human neutrophils were stimulated for 3h with respective PE/PPE individual proteins or combinations **(A)** or control proteins **(B)**, and the levels of calprotectin were measured as described in the Materials and Methods section. Results are depicted as log2-fold change values of calprotectin levels. The data were analyzed using 1-way ANOVA followed by Dunnett correction. Statistical significance was determined (** p < 0.05*) and the mean ± SD is represented (n= 3).

**
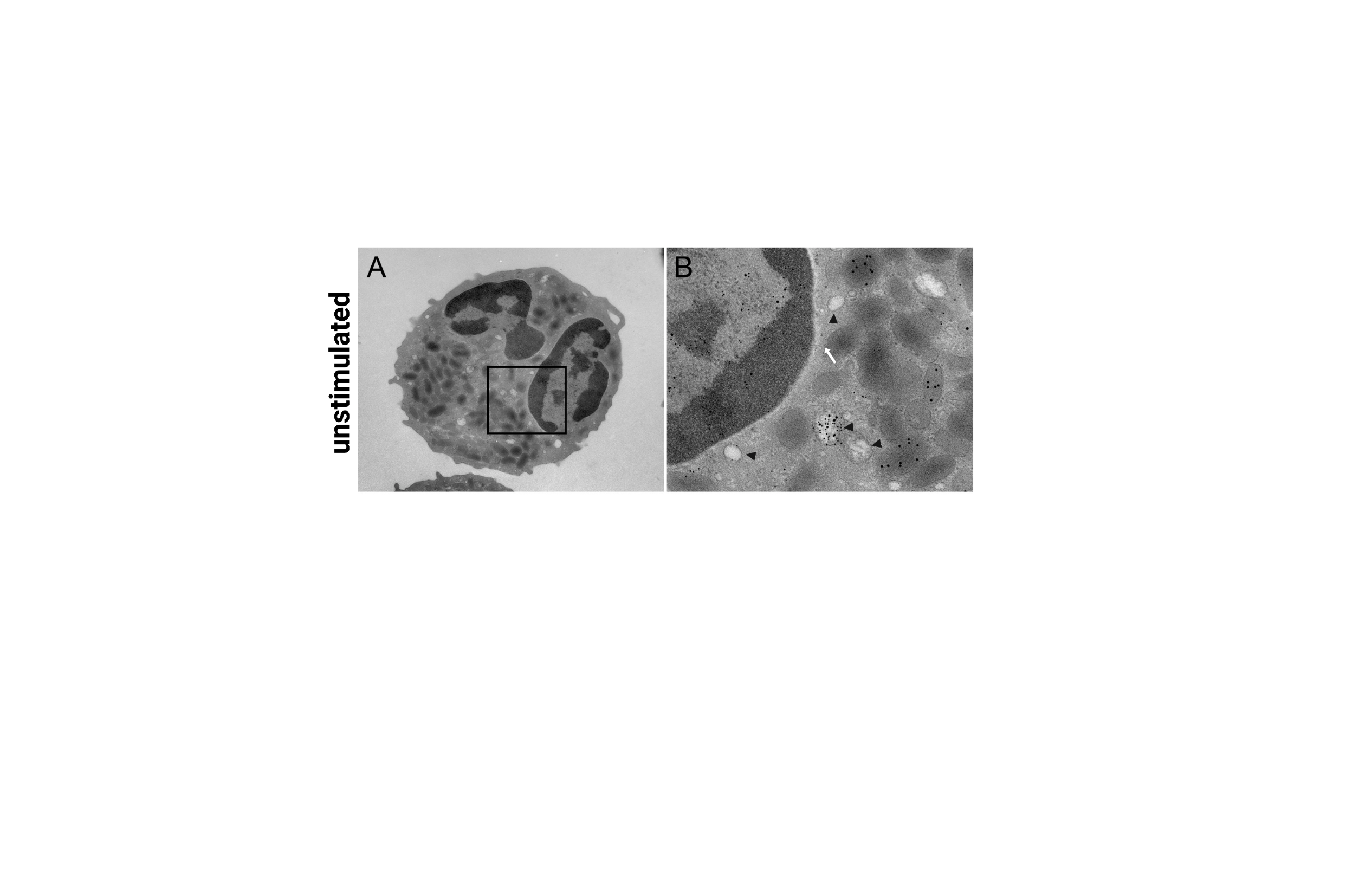
Supplementary Figure 6.** Representative TEM images of the control sample containing neutrophils unstimulated and incubated only in the presence of medium for 3h. (**A)** Overview of control neutrophil left unstimulated with respective zoom-in image **(B)** showing intact nucleus and presence of dense vesicles in the cytoplasm containing exclusively NE, as well as occasional organelles positive for both NE and H3cit.

Sections were stained with uranyl acetate and lead citrate (see Materials and Methods). 5 nm gold labeling= H3-cit; 10 nm gold labeling= NE. Scale bars in cell overview pictures= 2μm; zoom pictures= 250nm.
